# *Ruta* Essential Oils: Composition and Bioactivities

**DOI:** 10.3390/molecules26164766

**Published:** 2021-08-06

**Authors:** Lutfun Nahar, Hesham R. El-Seedi, Shaden A. M. Khalifa, Majid Mohammadhosseini, Satyajit D. Sarker

**Affiliations:** 1 Laboratory of Growth Regulators, Institute of Experimental Botany ASCR & Palacký University, Šlechtitelů 27, 78371 Olomouc, Czech Republic; 2 Biomedical Centre (BMC), Pharmacognosy Group, Department of Pharmaceutical Biosciences, Uppsala University, Box 591, SE-751 24 Uppsala, Sweden; hesham.el-seedi@farmbio.uu.se; 3 Department of Molecular Biosciences, The Wenner-Gren Institute, Stockholm University, SE-106 91 Stockholm, Sweden; shaden.khalifa.2014@gmail.com; 4 Department of Chemistry, College of Basic Sciences, Shahrood Branch, Islamic Azad University, Shahrood, Iran; majidmohammadhosseini@yahoo.com; 5 Centre for Natural Products Discovery (CNPD), School of Pharmacy and Biomolecular Sciences, Liverpool John Moores University, James Parsons Building, Byrom Street, Liverpool L3 3AF, UK

**Keywords:** *Ruta*, Rutaceae, *Ruta angustifolia*, *Ruta chalepensis*, *Ruta graveolens*, *Ruta montana*, *Ruta tuberculate*, essential oils, 2-nonanone, 2-undecanone, bioactivity

## Abstract

*Ruta* L. is a typical genus of the citrus family, *Rutaceae Juss*. and comprises ca. 40 different species, mainly distributed in the Mediterranean region. *Ruta* species have long been used in traditional medicines as an abortifacient and emmenagogue and for the treatment of lung diseases and microbial infections. The genus *Ruta* is rich in essential oils, which predominantly contain aliphatic ketones, e.g., 2-undecanone and 2-nonanone, but lack any significant amounts of terpenes. Three *Ruta* species, *Ruta chalepensis* L., *Ruta graveolens* L., and *Ruta montana* L., have been extensively studied for the composition of their essential oils and several bioactivities, revealing their potential medicinal and agrochemical applications. This review provides a systematic evaluation and critical appraisal of publications available in the literature on the composition and bioactivities of the essential oils obtained from *Ruta* species and includes a brief outlook of the potential applications of nanotechnology and chitosan-based products of *Ruta* essential oils.

## 1. Introduction

The genus *Ruta* L. belongs to the tribe Ruteae of the family *Rutaceae Juss*. and comprises ca. 40 different accepted species, which are native to or naturalized in many countries worldwide, especially in African, Asian, and European countries, e.g., Algeria, China, Iraq, Italy, Libya, Morocco, Portugal, Spain, Syria, and Tunisia, and have been introduced in the countries of North and South America [1,2]. However, the greatest distribution of *Ruta* species is found in the Mediterranean region. It can also be noted that the number of *Ruta* species as claimed by various authors may vary from as few as eight to as many as 160 species. *Ruta chalepensis* L., *Ruta graveolens* L., and *Ruta montana* L. are the three most widely distributed and most extensively studied species of the genus *Ruta*.

**Figure 1:**
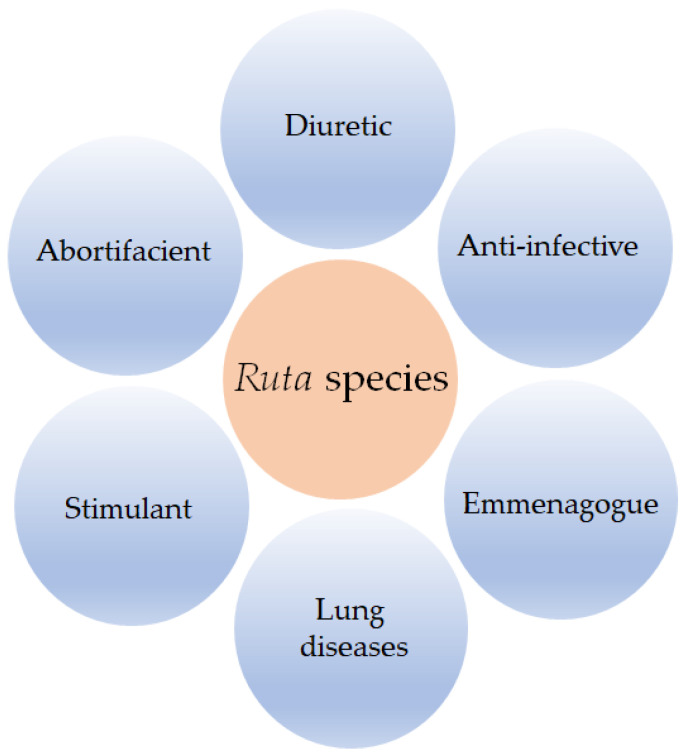
Major medicinal applications of *Ruta* species in traditional medicines.

The use of *Ruta* species in traditional medicines can be traced as far back as the 5th century BCE as documented by various famous authors like Hippocrates and Dioscorides [3], and the potential traditional medicinal value of these species is still appreciated in many countries as is evident from their continued use in the Ayurvedic, Unani, and Siddha medicines. In fact, various species of this genus have long been included in the European Pharmacopoeia. Traditional medicinal uses of *Ruta* species include their uses mainly as an abortifacient and emmenagogue and in the treatment of lung diseases and microbial infections (Figure 1) [3,4]. Moreover, *Ruta* species have the potential for use as a potassium channel blocker in the treatment of neuromuscular diseases, anxiety, and dysmenorrhea. In addition to the presence of several bioactive alkaloids, coumarins (especially furanocoumarins), and flavonoids (mainly quercetin and its derivatives) in *Ruta* species, many of which are chemotaxonomically characteristic of the family Rutaceae, the species of this genus are also rich in essential oils, which contribute to their aromatic, agrochemical, and medicinal properties [4,5,6]. This review systematically evaluates and critically appraises the literature available to date on the composition and bioactivities of the essential oils obtained from *Ruta* species and includes a brief overview of the potential applications of nanotechnology and chitosan-based products of *Ruta* essential oils.

## 2. Composition of *Ruta* Essential Oils

*Ruta* species are aromatic plants, and the relevant aromaticity is offered by the essential oils they produce. Essential oils are often a complex mixture of several compounds of various chemical classes, e.g., monoterpenes, sesquiterpenes, aliphatic alcohols, ketones, aldehydes, acids, and simple benzenoids. Generally, gas chromatography-flame ionization detection (GC-FID), gas chromatography-mass spectrometry (GC-MS), and gas chromatography-tandem mass spectrometry (GC-MS/MS) analytical techniques are used for the separation and identification of individual components present in essential oils, and these techniques provide qualitative and quantitative information required for the chemical profiling and quality control of essential oils [7].

**Figure 2:**
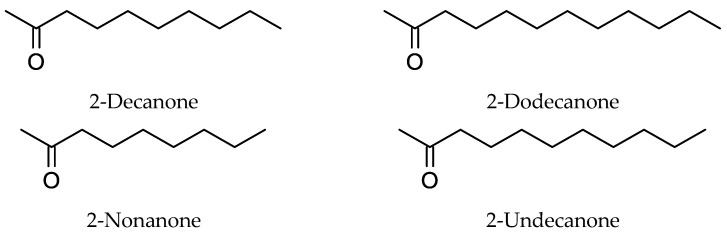
Major long-chain aliphatic ketones found in the *Ruta* essential oils.

From the studies on the composition of the essential oils of *Ruta* species, it appears that these essential oils, unlike many other plant essential oils, generally lack terpene components, but predominantly possess aliphatic ketones and some minor aliphatic alcohols. Based on the studies conducted on the dried or fresh plant materials of *Ruta* species (Table 1) [8,9,10,11,12,13,14,15,16,17,18,19,20,21,22,23,24,25,26,27,28,29,30,31,32,33,34,35,36,37,38,39,40,41,42,43,44,45,46,47,48,49,50,51,52,53,54,55,56,57,58,59,60,61,62,63,64,65,66,67,68,69,70,71,72,73], it is apparent that the long-chain aliphatic ketones, e.g., 2-undecanone (most abundant), 2-nonanone, 2-dodecanone, and 2-decanone (Figure 2) are the major components of *Ruta* essential oils. However, there are a few exceptions reported in the literature, for example, the air-dried aerial parts of *R. graveolens* from China were found to contain long-chain aliphatic esters like 2-undecanol acetate (19.2%) and 2-undecanol 2-methylbutyl ester (8.9%) [8], and the air-dried leaves of *R. chalepensis* from Beja (Tunisia) to possess menthol (43.9%), linalool (42.1%), and 2-hexanal (5.8%) as major components of the essential oil [9]. Similarly, terpenes like limonene and β-caryophyllene have also been found in reasonable amounts in some *Ruta* samples, e.g., the essential oils from *R. chalepensis* samples collected in Italy and Morocco showed the presence of limonene (1.8–12.8%) [10,11,12,13] and β-caryophyllene (~3.4%) was reported from the *R. graveolens* essential oil from Peru [14] and from the essential oil of *R. montana* collected in Algeria [15].

In fact, because of the common occurrence of 2-undecanone as the major compound in the essential oils of *Ruta* species, this compound has been indicated as a suitable chemotaxonomic marker for the genus *Ruta* L. [15]. However, for the same reason, 2-nonanone could also be another marker compound for the chemotaxonomic study of the genus *Ruta*.

**Figure 3:**
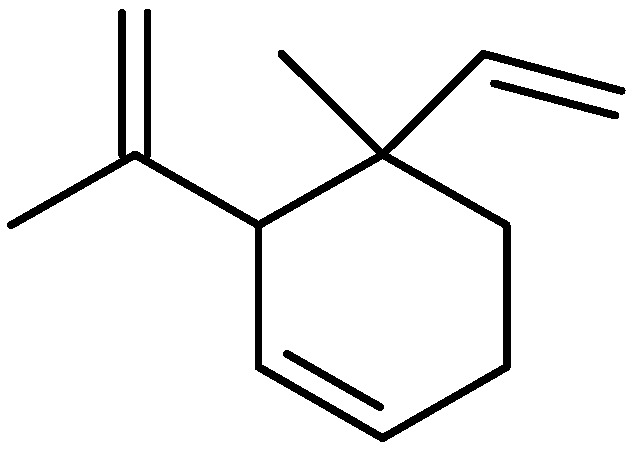
Structure of geijerene.

Most of the studies to date have been carried out with the whole aerial parts (fresh or dried), without differentiating flowers, fruits, leaves, and stems. However, there are a handful of reports (Table 1) available on the investigation of essential oils of individual parts/organs of *Ruta* species to understand the comparative chemical profiles and extraction yields. The roots of *Ruta* species have somehow been neglected as a source of any essential oil, but a genetically transformed root sample of *R. graveolens* was found to produce a percentage yield of 0.23% essential oil and to possess geijerene (67.0%) (Figure 3) as the major compound in its essential oil [16], and the root essential oil of *R. chalepensis* grown in Poland was found to biosynthesize octyl acetate, methyl decanoate, and phytyl acetate as major components [17].

The essential oils of *R. chalepensis*, including its subspecies and varieties, e.g., *R. chalepensis* subsp. *angustifolia* (Pers.) P. Cout., *R. chalepensis var. bracteosa* (DC) Boiss., and *R. chalepensis* subsp. *latifolia* (Salisb.) Linds. [10,11,15,17,18,19,20,21,22,23,24,25,26,27,28,29,30,31,32,33,34,35,36,37,38,39,40,41,42,43,44,45,46,47,48,49], and another species, *R. graveolens* [8,16,18,50,51,52,53,54,55,56,57,58,59,60,61,62,63,64,65,66] collected from different parts of the world have been extensively investigated, and considerable variations have been observed both in the yield (%) of essential oils, and their qualitative and quantitative chemical profiles (Table 1). Nevertheless, there are a good number of reports on the analysis of essential oil produced by *R. montana* (Table 1) [41,62,67,68,69,70,71,72,73]. There appears to be only one report each on the essential oil analysis of the species, *R. angustifolia* Pers. and *R. tuberculata* Forssk. [18]. However, *R. angustifolia* Pers. may actually be one of the subspecies of *R. chalepensis* rather than a separate species.

There have been significant inter- and intra-species differences observed in the yield (%) and major composition of the essential oils of the *Ruta* species (Table 1). Differences in chemical profiles and yields of the *Ruta* essential oils have been observed because of the differences in geographical origins, collection times (e.g., flowering and post-flowering stages), climatic conditions, seasonal variations, processing methods, stress level, and extraction protocols [15,40,51,54,55]. *Ruta* samples collected even in a single country, but from different locations, showed noticeable differences in yields and composition of the essential oils. For example, the essential oils of *Ruta* samples collected from different locations in Tunisia demonstrated considerable differences both in % yield and chemical composition [9,36,37,38,39,40,61,62] (Table 1), and a similar observation could be made for the *R. montana* samples collected from various sparts of Algeria [67]. Considerable variations in the essential oil yields (%) and chemical profiles have also been observed between cultivated and wild-growing plant materials from *Ruta* species [44]. However, hardly any difference in % yield of essential oils in the leaves of wild-grown and micropropagated samples of *R. graveolens* (0.80% and 0.84%, respectively) could be observed [57].

## 3. Bioactivities

Essential oils are well-known for their bioactivities, e.g., antimicrobial and antioxidant, and have long been used in different traditional medicine systems as well as in modern medicine, cosmetics, and pharmaceutical products [29,74,75,76,77,78,79,80,81,82,83,84,85,86,87,88,89,90,91,92]. Their applications as an insecticidal, larvicidal, nematocidal, pesticidal, phytotoxic, and insect-repellent agents are also well-documented [93,94,95,96,97,98,99,100,101,102,103,104,105,106,107,108,109,110,111,112,113,114,115,116]. *Ruta* essential oils found their way into various traditional medicinal products, and a significant body of literature could establish their bioactivities and medicinal potential [4]. Major bioactivities of *Ruta* essential oils include anti-inflammatory, antimicrobial, antioxidant, antiprotozoal, cytotoxic, herbicidal, insecticidal, insect repellent, larvicidal, nematocidal/anthelmintic, and phytotoxic activities. Significant differences in potencies in bioactivities do exist among different *Ruta* species because of the variations in the chemical composition of the essential oils arising from *Ruta* species being collected from different geographical sources, at different collection times, and from different plant parts, e.g., leaves, stems, flowers, fruits, and roots. The following subsections capture these bioactivities with specific examples.

### 3.1. Antimicrobial Activity

Plant essential oils, e.g., tea tree, rosemary, and lavender oils, have long been known for their antimicrobial properties [77]. The antimicrobial properties of *Ruta* essential oils are also known [4]. *Ruta* essential oils, aliphatic ketones being the major components, have been reported to possess weak to moderate levels of antibacterial and antifungal properties compared to standard antibiotics [4,78,79]. By virtue of these properties, *Ruta* essential oils and their components, e.g., 2-undecanone, 2-undecanol, and their derivatives, have been used in various antimicrobial traditional medicine preparations, and in the prevention of decay of fruits and vegetables to enhance their shelf-life.

**Figure 4:**
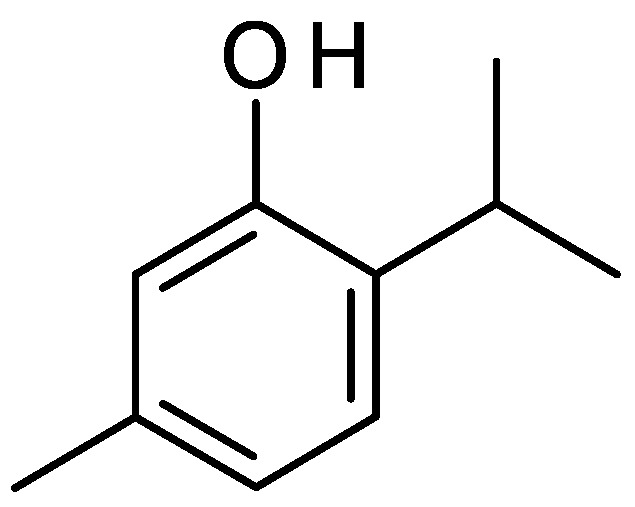
Structure of 2-isopropyl-5-methylphenol.

Most of the in vitro antimicrobial studies reported to date on *Ruta* essential oils were performed with total essential oils and using the classical agar disc diffusion assay or microdilution assay. However, the most abundant compound of the *Ruta* essential oils, 2-undecanone (Figure 2), and its derivatives were tested for their antimicrobial property against two bacterial species, namely, *Bacillus subtilis* and *Escherichia coli*, and two fungal species, *Aspergillus niger* (mold) and *Candida mycoderma* (yeast) using the impedimetric method [80]. 2-Undecanone showed a low level (MIC = >30 μL/mL) of bacteriostatic activity against both bacterial species but was more active against the fungal species (MIC 1 μL/mL). The antibacterial potency of 2-isopropyl-5-methylphenol (Figure 4), isolated from the essential oil from the aerial parts of *R. graveolens*, and several of its analogs were evaluated against three Gram-positive (*Bacillus cereus*, *Listeria monocytogenes*, and *Staphylococcus intermedius*), and three Gram-negative (*Salmonella enterica*, *Salmonella typhimurium*, and *Shigella sonnei*) bacterial species [59], and 2-isopropyl-5-methylphenol (Figure 4) showed significant antibacterial activity (zones of inhibition 18–20 mm) against all six tested bacterial species, with the positive control tetracycline having zones of inhibition of 20–24 mm. The antibacterial and antifungal activities of *Ruta* essential oils are described with specific examples in the following subsections.

#### 3.1.1. Antibacterial Activity

*Ruta* essential oils, mainly from *R. chalepensis*, *R. graveolens*, and *R. montana*, have been shown to possess low to moderate levels of antibacterial activity against both Gram-positive and Gram-negative bacteria [4,79] (Table 2). In most of the antibacterial screenings of *Ruta* essential oils, a standard mix of 10–12 Gram-positive and Gram-negative human pathogenic bacterial species from the genera *Bacillus*, *Citrobacter*, *Enterococcus*, *Escherichia*, *Klebsiella*, *Listeria*, *Proteus*, *Pseudomonas*, *Salmonella*, and *Staphylococcus* was used. In some studies, however, plant pathogenic bacterial species like *Clavibacter michiganensis* subsp. *michiganensis* and *Xanthomonas albilineans* were used [23]. The levels of activities among the essential oils from the genus *Ruta* were found to vary significantly, both in potency and susceptibility (Table 2). No preferential or selective antibacterial activity could be observed between Gram-positive and Gram-negative groups. Antibacterial activities reported in the literature are from routine assays and are of a preliminary nature, without any reasonable efforts towards establishing the plausible mechanisms of action or identifying components responsible for the activity.

The essential oil obtained from the aerial parts of *R. angustifolia* was found to be inactive against most of the tested organisms but was active against *Bacillus cereus* (10 mm), *Enterococcus faecalis* (8 mm), and *Citrobacter freundii* (7 mm) [18]. It should be mentioned that the disc diameter was 6 mm, and any activity mentioned with a zone of inhibition of 6 mm should be considered inactive. The antibacterial activity reported for *R. chalepensis* essential oil was mostly associated with the essential oils obtained from its whole aerial parts and leaves (Table 2). The only report on the antibacterial activity of its root essential oil was observed against *Listeria monocytogenes* and *Pseudomonas aeruginosa* [17], while that of the essential oil from its stem was tested against *Escherichia coli*, *Listeria monocytogenes*, *Pseudomonas aeruginosa*, *Salmonella typhi*, and *Staphylococcus aureus* [17]. This study [17] demonstrated that a significant difference could exist between the antibacterial profiles of *Ruta* essential oils obtained from two different organs of the same plant and that they can have different chemical properties. The essential oil from the aerial parts of *R. chalepensis* L. *var. bracteosa* was active against *Acinetobacter baumanii* (12 mm), *Bacillus cereus* (12 mm), *Enterococcus faecalis* (10 mm), *Citrobacter freundii* (7 mm), *Escherichia coli* (7 mm), *Proteus mirabilis* (10 mm), *Salmonella typhi* (15 mm), and *Staphylococcus aureus* (17 mm), while no activity was observed against *Citrobacter freundii*, *Enterobacter cloacae*, *Klebsiella pneumoniae*, *Listeria monocytogenes*, and *Pseudomonas aeruginosa* [18].

Like *R. chalepensis*, the essential oils obtained from the whole aerial parts and leaves of *R. graveolens* have mainly been explored for their potential antibacterial activity (Table 2). The study conducted by Owlia et al. [82] could not find any detectable activity of the essential oil from the aerial parts of *R. graveolens* against *Pseudomonas aeruginosa*. Reddy et al. [56] reported a moderate level of activity with a zone of inhibition of 15 mm against this species. In fact, the work carried out by Reddy et al. [56] with the essential oils from the aerial parts of *R. graveolens* is one of the most extensive in vitro antibacterial activity assessments to date, and this study revealed activity against a range of bacterial species, e.g., *Acinetobacter baumannii* (19 mm), *Bacillus cereus* (28 mm), *Citrobacter freundii* (16 mm), *Enterobacter aerogenes* (13 mm), *Enterobacter cloacae* (18 mm), *Enterococcus faecalis* (27 mm), *Escherichia coli* (18 mm), *Klebsiella pneumoniae* (18 mm), *Listeria monocytogenes* (18 mm), *Micrococcus flavus* (21 mm), *Micrococcus luteus* (19 mm), *Proteus mirabilis* (22 mm), *Pseudomonas aeruginosa* (15 mm), *Salmonella typhimurium* (13 mm), and *Staphylococcus aureus* (23 mm). They not only reported the zones of inhibition against the tested bacterial species, but also worked out the MIC values (0.65–1.58 μg/mL in most cases) (Table 2). While the anti-*Helicobacter pylori* activity of this essential oil was reported in one study [83], the activity against *Legionella pneumophila* (MIC < 0.02–0.40 μg/mL) was only reported by Chaftar et al. [65]. In addition to activity against human pathogenic bacterial species, Mahmoud et al. [54], assessed the antibacterial property of the essential oil from the leaves of *R. graveolens* against plant pathogenic bacterial species *Dickeya solani* (420 μg/mL), *Pectobacterium atrosepticum* (310 μg/mL), and *Pectobacterium carotovorum* subsp. *carotovorum* (110 μg/mL). It can be noted that the bacterial genus *Pectobacterium* comprises several enterobacterial species that produce plant diseases, e.g., soft rot, wilt, and blackening in potato and ornamental plants. Similarly, *Dickeya solani* is also a causative bacterial species for soft rot and blackening of potato crops.

The essential oils from the whole aerial parts and leaves of *R. motana* have also been found to possess antibacterial properties (Table 2). Drioiche et al. [71] conducted probably the most elaborate antibacterial study on the essential oil of the aerial parts of this *Ruta* species, and found low–moderate levels of activity against, *Citrobacter koseri* (8 mm), *Corynebacterium* sp. (11 mm), *Enterococcus faecalis* (7 mm), *Enterococcus faecium* (17 mm), *Escherichia coli* (8 mm), *Klebsiella oxytoca* (8 mm), *Listeria* sp. (11 mm), *Proteus mirabilis* (7 mm), *Pseudomonas aeruginosa* (8 mm), *Salmonella* sp. (11 mm), *Serratia marcescens* (8 mm), *Staphylococcus aureus* (8 mm), *Staphylococcus haemolyticus* (8 mm), *Streptococcus acidominimus* (7 mm), *Streptococcus porcinus* (8 mm), and *Yersinia enterolitica* (8 mm), but no activity was reported against *Enterobacter aerogens*, *Enterobacter cloacae*, *Klebsiella pneumonie ssp. Pneumonie*, *Pseudomonas fluorescence*, *Pseudomonas putida*, *Shigella* sp., *Staphylococcus epidermidis*, *Streptococcus agalactiae*, and *Streptococcus groupe.* In another study, the essential oil of the aerial parts was found to be inactive against *Escherichia coli* but showed significant activity against *Staphylococcus aureus* with a zone of inhibition of 18 mm [70].

The only report on the antibacterial activity of the essential oil from the aerial parts of *R. tuberculata* revealed activity against *Bacillus cereus*, *Enterococcus faecalis*, *Salmonella typhi*, and *Staphylococcus aureus* with zones of inhibition between 8 and 14 mm (Table 2), but no activity was observed against *Acinetobacter baumanii*, *Citrobacter freundii*, *Enterobacter cloacae*, *Escherichia coli*, *Klebsiella pneumoniae*, *Listeria monocytogenes*, *Proteus mirabilis*, or *Pseudomonas aeruginosa* [18].

#### 3.1.2. Antifungal Activity

*Ruta* essential oils were shown to possess significant levels of antifungal activity, fungicidal as well as fungistatic, against several common fungal species, e.g., *Alternaria alternata*, *Aspergillus fumigatus*, *Aspergillus niger*, *Candida albicans*, and *Fusarium oxyxporum* (Table 3), and in certain cases, the activity was comparable to or even better than the positive control (known antifungal agents, e.g., amphotericin) [4,18,84,85] (Table 3). The essential oil from the aerial parts of *R. angustifolia* showed the most potent antifungal activity against *Candida albicans* (35 mm) and the least activity against *Cladosporium herbarum* (18 mm), while that of *R. chalepensis var. bracteosa* was most active against *Cladosporium herbarum* (35 mm) and the least against *Fusarium oxysporum* (8 mm) [18]. *Ruta* essential oils not only demonstrated activity against human pathogenic fungal species, but showed activity against plant pathogenic fungi as well, for example, the essential oil of the aerial parts of *R. chalepensis* appeared to be active against *Alternaria* sp. [40,86]. Although *Alternaria* sp. are generally plant pathogens, they are common allergens and cause hay fever and hypersensitivity reactions in humans. Generally, the essential oils of *Ruta* species, especially *R. chalepensis*, have been found to be effective against various *Aspergillus* species [17,18,42,87].

The essential oil of the aerial parts of *R. graveolens* exhibited antifungal activity against *Melassezia furfur*, which is usually found on the human skin and is responsible for skin diseases and dermatological conditions [88]. This essential oil was also active against several *Candida* species, namely, *C. albicans* (MIC 8.2 μg/mL), *C. parapsilopsis* (MIC 16.4 μg/mL), *C. glabrata* (MIC 4.1 μg/mL), and *C. tropicalis* (MIC 131.0 μg/mL) [89], and the plant pathogenic fungi *Sclerotinia sclerotiorum* (which causes white mold disease in plants) [90], *Bipolaris oryzae*, and *Gerlachia oryzae* [91,92].

The major antifungal activity of the essential oil of the aerial part of *R. montana* was against *Aspergillus*, *Alternaria*, and *Candida* species [18,67,70,71,72,73,84]. This essential oil was also found to be active against *Saccharomyces cerevisiae* (12–15 mm) [67]), *Cryptococcus neoformans* (20 mm), *Fusarium* sp. (14 mm), *Penicillium* sp. (15 mm), *Rhodotorula rubra* (11 mm), *Trichophyton mentagrophytes* (15 mm), and *Trichosporon* sp. (17 mm) [71], as well as *Botrytis cinerea*, *Fusarium oxysporum*, *Fusarium solani*, and *Verticillium dahlia* with MICs 100–1100 μg/mL [18,73]. A summary of the antifungal activities of *Ruta* essential oils is presented in Table 3.

### 3.2. Antioxidant Activity

Oxidative stress is often implicated in several chronic and severe illnesses like diabetes, cancers, cardiovascular diseases, and many more. External supply of antioxidants, either as fresh fruits and vegetables or as therapeutic and preventative commercially available pharmaceutical and/or nutraceutical products, may be necessary to mitigate oxidative stress. Antioxidants are compounds, synthetic (e.g., BHT (butylated hydroxytoluene), BHA (butylated hydroxyanisole) and propyl gallate) or natural (e.g., carotene, ascorbic acid, quercetin, and resveratrol), that can mitigate oxidative stress by virtue of their free-radical-scavenging ability and/or reducing power. The search for new, safe, and effective antioxidants from natural origins has intensified in recent years. As plant essential oils have been shown to possess various degrees of antioxidant capacities, mainly because of the presence of various simple phenolic compounds, e.g., carvacrol, coniferyl alcohol, eugenol, gualacol, and thymol, and have been exploited effectively for their uses as food preservatives and in nutraceutical products [93,94], the volume of research aiming at establishing the antioxidant properties of essential oils from new plant sources and their components has also increased.

As part of this relatively new popular trend in phytochemical research, *Ruta* essential oils have been investigated for antioxidant activity by various researchers. One of the early investigations into the antioxidant properties of *Ruta* species was conducted by Kambouche et al. [69] on the essential oil from the aerial parts of *R. montana* from Algeria using the DPPH (2,2-diphenyl-1-picryhydrazyl) radical scavenging assay, where a concentration-dependent antiradical activity was observed. Later, essential oil obtained from the aerial parts of this *Ruta* species collected from the Middle Atlas Mountains, Morocco, was assessed for radical-scavenging activity using the same assay, and the IC_50_ value was determined as 548.5 μg/mL compared to that of the positive control ascorbic acid (vitamin C) at 53.4 μg/mL [71]. The radical-scavenging activity was at least two-fold more potent than that of the hydromethanolic extract and the hydromethanolic macerate of the aerial parts.

The same DPPH radical-scavenging assay was used to determine the radical-scavenging property of the essential oil from the aerial parts of *R. chalepensis* from Tunisia, and a low level of activity (0.6–5.61%) at a concentration of 200 mg/mL was detected, which was much less potent than that of the synthetic antioxidant BHT [95]. The essential oils from the Palestinian samples of *R. chalepensis* were investigated for their antioxidant potential solely based on the DPPH assay [33], and a concentration-dependent radical-scavenging activity for all samples was observed, with some differences in activity among the samples collected from three different places, Jerusalem, Hebron, and Jenin, having IC_50_ values of 6.9, 7.8, and 19.9 μg/mL, respectively. The radical-scavenging activity was assumed to be linked to high concentrations of linalyl acetate present in the essential oils. Coimbra et al. [4] and Malik et al. [79] documented the antioxidant property of the essential oils obtained from different plant parts of *R. graveolens*. However, none of these studies was comprehensive enough to establish the antioxidant potential of the essential oils, as the DPPH assay can only reveal the radical-scavenging property, which is one of the several mechanisms for antioxidant activity. For a conclusive and comprehensive understanding of the antioxidant property of any sample, a battery of antioxidants assays, e.g., reducing capacity assay, bleaching assay, scavenging of other radicals, enzymatic assays, etc., should be applied.

A combination of the DPPH assay and the ABTS [2,2′-azinobis-(3-ethylbenzothiazoline-6-sulfonate)] radical-scavenging assay was utilized for the determination of the antioxidant potential of the essential oil from the leaves of *R. chalepensis* from Tunisia, and interestingly, a higher radical-scavenging activity was observed with this essential oil than that of the methanolic extract, which normally contains various well-known phenolic and polyphenolic antioxidants [9]. It was suggested that the strong radical-scavenging property of this essential oil could be due to the presence of linalool and menthol. The essential oils from the stems and roots of this plant were also tested, but the potency was much less than that of the essential oil obtained from the leaves. Later, Althaher et al. [28] assessed the antioxidant activity of the essential oil from the aerial parts of the Jordanian *R. chalepensis* using a combination of the DPPH assay and the reducing power assay. Their work demonstrated a significant radical-scavenging property (IC_50_ 35 μg/mL) compared to that of the positive control ascorbic acid (IC_50_ 21.2 μg/mL). A dose-dependent reducing power capacity was observed with this essential oil, and the EC_50_ (effective dose 50%) was calculated as 20110 μg/mL compared to 90.6 μg/mL for ascorbic acid, indicating its extremely weak reducing power capacity compared to ascorbic acid. It is known that ascorbic acid is more potent as a reducing agent than as a radical-scavenger, and for the DPPH assay, it is advisable to use a more potent radical-scavenger like quercetin or gallic acid as a positive standard.

Mohammedi et al. [67] used the DPPH and the reducing power assays to determine the antioxidant activity of the essential oils from the aerial parts of *R. montana* collected from different locations in Algeria. In the DPPH assay, the IC_50_ (inhibition concentration 50%) values for the samples were in the range from 49.6 to 68.1 μg/mL, while the IC_50_ value representing the reducing power was 46.0–64.4 μg/mL. It is interesting to note that the differences in the antioxidant potency among the samples, as determined by the DPPH assay and reducing power assay, were comparable and followed the same pattern, e.g., the most potent radical-scavenging sample as revealed by the DPPH assay also displayed the most potent reducing power. Earlier, a similar approach, combining the DPPH and reducing power assays, was adopted by Benali et al. [72] to assess the antioxidant potential of the essential oil of the aerial parts of *R. montana*, collected from Taza, Morocco, and the IC_50_ value for the DPPH radical-scavenging ability was found to be 244.6 μg/mL. The reducing power of this essential oil, expressed in milligram equivalence of ascorbic acid per gram of essential oil, was 1.39 mg/g.

None of the studies published for *Ruta* essential oils assessing their antioxidant potentials appear to have attempted any in vivo studies, enzyme assays, or tried to establish conclusively which components of the essential oils could be responsible for the antioxidant activity. Clearly, much more work is needed if any of the *Ruta* essential oils are to be considered for commercial uses as natural antioxidants.

### 3.3. Anti-Inflammatory Activity

While the anti-inflammatory activity of various extracts of *Ruta* species, particularly *R. graveolens*, has been well-documented in the literature [4,79], the anti-inflammatory activity of *Ruta* essential oils has not been studied adequately to date. The anti-inflammatory activity of the essential oils of *R. chalepensis* of Algerian origin was evaluated by the carrageenan-induced paw edema method using the *albino* mice [20]. It was found that this essential oil at a dose of 0.5 mL/mouse could significantly reduce carrageenan-induced edema as well as the positive control, dichlofenac. It was assumed that the anti-inflammatory activity of this essential oil could be mediated through the inhibition of inflammation mediators like serotonin, prostaglandin, and histamine. Earlier, a similar anti-inflammatory effect was reported for the essential oil obtained from the aerial parts of *R. graveolens* [96]. None of these studies attempted to establish a link between the composition and the anti-inflammatory property of the *Ruta* essential oils.

It is well-known that oxidative stress can activate several transcription factors, leading to the differential expression of some genes involved in inflammatory pathways, and thus, any effective reduction in oxidative stress by antioxidants or free-radical-scavengers is expected to result in a reduction in inflammatory responses [97]. As *Ruta* essential oils have been shown to possess considerable antioxidant or free-radical-scavenging potential as outlined above, it is reasonable to assume that further anti-inflammatory activity studies will potentially reveal anti-inflammatory properties of *Ruta* essential oils, their components, and their applications in the management of inflammatory diseases and inflammation in general.

### 3.4. Antiparasitic Activity

Parasites are organisms that live and feed on another living being, e.g., animals, humans, insects, or plants, and most often cause harm to the host organism. One of the major traditional medicinal uses of *R. chalepensis*, and other *Ruta* species in general, is as antiparasitic agents [5], particularly as an anthelmintic medication. In fact, from an ethnopharmacological survey, it was obvious that extracts and essential oils of *Ruta* species, particularly *R. graveolens*, have long been used as an antiparasitic agent in traditional medicine in Italy [98]. This traditional medicinal use of *R. chalepensis* has prompted antiparasitic activity screening of its essential oils, extracts, and isolated major compounds. The antiparasitic activity of plant essential oils is well-documented in the literature [99], and *Ruta* essential oils have been investigated for their possible role as natural antiparasitic agents [4]. A study conducted by Castagnino and Orsi [99] demonstrated the efficacy of the essential oil of *R. graveolens* on the control of *Varroa destructor* (mite) infestation in bee (*Apis melifera*) colonies. It was found that the essential oil of *R. graveolens* could significantly affect mite mortality levels and the mite infestation rate, and could reduce bee offspring mortality rate by 83.3%. It can be noted that *V. destructor* of the family Varroidae is an external parasitic mite that lives on the honeybee hosts, *Apis cerana* and *Apis melifera*.

**Figure 5:**
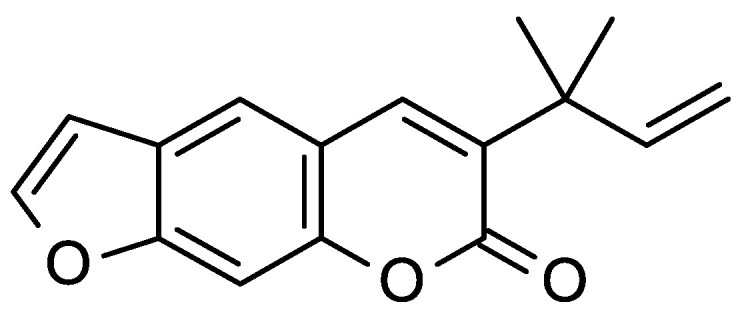
Structure of chalepensin.

A protozoa can be free-living or parasitic. Most of the disease-causing protozoa are parasitic. Even free-living protozoa, when they enter the cells or tissues of living beings, become parasitic. Antiprotozoal activity of chalepensin (Figure 5), a 3-prenylated furanocoumarin, isolated from the methanolic extract of the aerial parts of *R. chalepensis*, but also present in small amounts in some of its essential oils [36] (Table 1), was established against the parasite *Entamoeba histolytica*, which is the major cause of amoebiasis in humans [100]; 84.66% growth inhibition was observed against *E. histolytica* at a concentration of 150 µg/mL of chalepensin.

Leishmaniasis is a vector-borne parasitic disease caused by the obligate intracellular protozoic parasites of the genus *Leishmania*, which mainly affects people living in the tropics, subtropics, and southern Europe. A study conducted with 10 different plant essential oils from Tunisia examined the effectiveness of these oils as potential leishmanicidal agents against *Leishmania major* and *L. infantum* [95]. The 2-undecanone-rich (84.2%) essential oil of the aerial parts of *R. chalepensis* was found to be active only against *L. infantumi*, and interestingly, *L. major* promastigotes were resistant to this essential oil at a concentration of 8 μg/mL. Based on the outcome from the cytotoxicity assay, it was suggested that the leishmanicidal activity of this essential oil might not be completely associated with its cytotoxicity and that other mechanisms of action could be involved.

Although nematodes and various fungi are parasites living in/on animals, humans, or plants, the activities of *Ruta* essential oils as nematicides and antifungal agents are discussed under different sub-sections later in this review.

### 3.5. Cytotoxic Activity

Cancer is one of the major reasons for human mortality and morbidity. Currently available cancer treatment choices are rather limited, and often produce severe side effects and toxicities. Therefore, the search for new, effective, safe, and affordable anticancer drugs is part of many major modern drug discovery initiatives worldwide, and there are reports on studies looking at the potential of plant essential oils as cytotoxic agents against cancer cells. *Ruta* essential oils are not an exception, albeit the actual number of studies on cytotoxicity of *Ruta* essential oils is limited. Althaher et al. [28] studied the cytotoxicity of the essential oil of the aerial parts of *R. chalepensis* collected in Jordan using the MTT [3-(4,5-dimethylthiazol-2-yl)-2,5-diphenyltetrazolium bromide] assay against the MCF-7 (mammary gland carcinoma), T47D (human ductal breast epithelial cancer), Caco-2 (colorectal adenocarcinoma), and MRC-5 (normal human fibroblast) cell lines. A dose-dependent cytotoxic effect of this essential oil was observed against all tested cancer cell lines with IC_50_ values ranging between 79.3 and 107.7 μg/mL, but no cytotoxicity was noted against the normal human fibroblasts (MRC-5). The essential oil was the most cytotoxic to MCF-7 and the least to Caco-2 cell lines. However, no inference to any specific components of the essential oil that might be responsible for this cytotoxicity was made. A low level of cytotoxicity of the essential oil of *R. chalepensis* was previously demonstrated against the murine macrophage cell line RAW264.7 [95].

Antiproliferative, apoptotic, and caspacse-3/7 activities of the essential oil of *R. graveolens* and its major components were observed against several human cell lines, e.g., MCF-7, HeLa (Henrietta Lacks cervical cancer), Jurkat (T lymphocyte), T24 (transitional cell carcinoma), HF-19 (human fetal lung fibroblasts), and HEK-293 (human embryonic kidney), and the activity was enhanced with the essential oil obtained from mixture treated plants [54]. For example, the IC_50_ of the untreated essential oil against MCF-7 was 20.1 μg/mL, whereas with seaweed *(Ascophyllum nodosum)* treated essential oil, it was 15.0 μg/mL. In the antiproliferation and apoptosis assay, the IC_50_ values for the untreated and treated (with *Ascophyllum nodosum*) essential oils (IC_50_ values for untreated sample are in parentheses) against other cell lines, HeLa, Jurkat, T24, HF-19, and HEK-293 were 6.5 (5.2), 26.0 (19.5), 95.3 (83.9), 122.2 (90.1), and >200 (>200) μg/mL, respectively. The IC_50_ values for 2-undecanone, one of the major components of this essential oil, against the above cell lines were, respectively, 4.2, 2.1, 8.3, 41.5, 33.3, and >200 μg/mL, while that of the other major compound, 2-nonanone, were 7.1, 3.2, 14.2, 64.1, 57.3, and >200 μg/mL, respectively. Cytotoxicity of the essential oil of *R. graveolens* was also studied against human lymphocytes maintained in culture and the mutagenicity was assessed by the Ames test with the *Salmonella typhimurium* strain TA-100 [83]. Although the concentrated essential oil showed some cytotoxicity (40% lower cell viability), on subsequent dilutions, it appeared to have lost its cytotoxicity and mutagenicity, suggesting its safety, in low amounts, for human consumption as a medicine.

### 3.6. Herbicidal Activity

Herbicides are compounds or extracts that can kill herbs (plants) or prevent their growth; they can be selective or non-selective. Because of the potential health hazards of herbicides currently available on the market, there has been a renewed interest in discovering new, cost-effective, and safer herbicides, not necessarily as purified compounds from natural sources, but also as crude extracts or essential oils. *Ruta* essential oils have been shown to possess herbicidal properties, and some examples of these properties are discussed below.

Bouajaj et al. [10] studied the in vitro efficacy of *R. chalepensis* essential oil for its herbicidal potency, and their influence on the germination of shoot and root growth of weeds, *Triticum durum* and *Phalaris canariensis*. Later, it was found that the herbicidal activity of the essential oils of *R. chalepensis* was dose-dependent and would depend on the weed species [40]. This essential oil could inhibit germination (90% rate at 2 μL/mL concentration) and seedling growth of weeds. The activity could be attributed to the presence of α-pinene and other oxygenated monoterpenes in the essential oil of *R. chalepensis*. An emulsion based on 6% essential oil of the aerial parts of *R. chalepensis* collected from El Fahs region, Tunisia, showed a sprouting inhibitory effect on potatoes [37]. The anti-sprouting effect of this essential oil was dose-dependent. It was found that the final weight of sprout for untreated sample was 4.66%, but the weight for the essential oil treated sample was only 0.98%. The major component of this essential oil, 2-undecanone, was thought to be responsible for this activity.

Faria et al. [60] demonstrated that the essential oil of the aerial parts of *R. graveolens* could completely inhibit the growth of the hairy roots of *Solanum tuberosum*. The herbicidal or phytotoxic property of *R. graveolens*, however, was reported previously on *Raphanus sativas*, where this essential oil inhibited the germination and seedling radicle growth [101]. In a recent study, the essential oil obtained from the fresh leaves of *R. graveolens* sourced from Brazil has been shown to significantly inhibit the germination and primary development of *Eragrostis plana* (lovegrass), which is well-known as an invasive plant, at a low concentration (0.01%), and the inhibition could be as much as 57.5% at a concentration of 0.1% [102]. In another study [66], the essential oils from the fresh flowers and leaves of this *Ruta* species were found to affect dose-dependently (at a concentration range 5–20 μL/mL) seed germinations and selling growth of *Amaranthus retroflexus* L., *Convolvulus arvensis* L., and *Rumex crispus* L., three well-known weed species in cultivated lands. At a concentration of 20 μL/mL, this essential oil could prevent seed germination completely (100%).

### 3.7. Insecticidal Activity

Plant essential oils are known to have insecticidal properties and have been employed in ecologically-friendly agricultural operations, including the control of pest insects of stored agricultural products [103]. *Ruta* essential oils are no exception and have been shown to possess insecticidal activity against various insect species, and have been proposed as natural and eco-friendly insecticides with the potential for replacing harmful conventional insecticides.

One of the earlier studies on the assessment of insecticidal properties of *Ruta* essential oils was carried out on *R. montana* essential oil, obtained from its fresh aerial parts collected in Tipaza, Algeria [68], and a clear dose-dependent insecticidal activity against German cockroaches (*Blatella germanica*) was reported. The highest impact (100%) was observed with an essential oil concentration of 1.6%. This oil was also found to be effective in killing mosquitoes *Culex pipiens* with 99% insecticidal effect at 0.6% essential oil concentration after 30 min. The impact of thionation of the essential oil of *R. montana* on insecticidal activity was explored by Fekhar et al. [70] using the fumigation toxicity assay employing *Sitophilus oryzae* adults. The non-thionated essential oil of *R. montana* could kill 13% of insects within 24 h of exposure, and thionation of the essential oil apparently resulted in an increase in insecticidal activity.

The essential oil of the aerial parts of *R. chalepensis* exhibited significant insecticidal activity against three major pests, coffee berry borer (also known as coffee borer beetle; *Hypothenemus hampei*; 82.5% mortality in 24 h), antestia bug (*Antestiopsis* sp.; 87.5% in 24 h), and maize weevil (*Sitophilus zeamais*; 73.5% in 24 h) [103]. Similar insecticidal activity of this essential oil, having 2-undecanone as the major component, was observed against two flour beetles, *Tribolium castaneum* and *T. confusum* in the contact toxicity assay, and the LD_50_ value was determined as being 0.09–0.13 μL/cm^2^ [32]. The insecticidal activity was attributed to the aliphatic ketone, 2-undecanone, which was previously reported to possess insecticidal properties [47]. Later, Akkari et al. [38] studied the insecticidal properties of the essential oils obtained from the flowers and leaves of *R. chalepensis*. More specifically, they assessed their larvicidal properties. In a recent study, essential oil from *R. chalepensis* growing in Morocco was shown to possess insect repellent and insecticidal properties against the adults of *Tribolium castaneum* [31]. It was found that, even at a low concentration, e.g., 0.15 μL/mL, the mortality rate was 55.56% after 48 h of exposure. A mortality rate of 57.9% could be achieved with a higher dose of 0.31 μL/mL after 24 h. A concentration-dependent insecticidal activity of the essential oil of *R. chalepensis* has been demonstrated further against lesser grain borer (*Rhyzopertha dominica*) using the fumigant toxicity assay [45]. Low mortalities (8.36, 11.22 and 15.96%) were observed, respectively, at 6.45, 11.29, and 16.13 µL/L (air), after 24 h. The mortality rate increased to 100% when a concentration of 129.03 µL/ (air) essential oil was applied.

The essential oils from the flowers and leaves of *R. graveolens* afforded an insecticidal compound, 2-isopropyl-5-methylphenol (Figure 4). Both the essential oil and the isolated compound were tested for fumigant toxicity as well as contact toxicity against stored-food pests (*Coleoptera* insects), e.g., *Lasioderma serricorne*, *Sitophilus zeamais*, and *S. oryzae* [104]. The LD_50_ values of this essential oil in the fumigant toxicity assay were, respectively, 0.480 and 0.527 mg/mL against *S. zeamais* and *S. oryzae*, while in the contact toxicity assay the values were 0.592 and 0.618 mg/cm^2^, respectively. No noticeable insecticidal activity of this essential oil was observed against *L. serricorne.* The LD_50_ values for the isolated compound, 2-isopropyl-5-methylphenol, in the fumigant toxicity were, 0.192 and 0.211 mg/mL, respectively, against *Sitophilus zeamais* and *S. oryzae*, and the values in the contact toxicity assay were 0.187 and 0.192 mg/cm^2^. There was no significant toxicity observed for this compound against *L. serricorne* in the fumigant toxicity assay, but it showed activity in the contact toxicity assay with an LD_50_ value of 0.398 mg/cm^2^. Ben Chaaban et al. [61] utilized the fumigant toxicity assay to assess the insecticidal potency of the essential oil obtained from *R. graveolens*. A dose-dependent insecticidal activity against *Ectomyelois ceratoniae* and *E. kuehniella* was observed with the essential oil, where mortality rate increased with the increase in concentration of the essential oil applied. For the lowest essential oil concentration (1.81 μL/L air), the percentage mortality of *Ectomyelois ceratoniae* after 48 h reached 4%, and 6% in the case of *E. kuehniella.* Similarly, for the highest concentration (54.54 μL/L air), the mortality rates were 62 and 88%, respectively, against *Ectomyelois ceratoniae* and *E. kuehniella* with this essential oil.

### 3.8. Insect-Repellent Activity

Several publications have reported the significant insect-repellent activity of *Ruta* essential oils. In a recent study, essential oil from *R. chalepensis* growing in Morocco was shown to possess insect-repellent properties against the adults of *Tribolium castaneum* as evaluated by the preferential area method on filter paper using the same conditions as those used for mass rearing (30 °C temp. and 70% humidity) [31]. At a dose of 0.038 μL/mL, this essential oil demonstrated 100% repellency after 15 min, and a dose of 0.031 μL/mL caused 100% repellency after 20 min of exposure. It was interesting to note that at a dose of 0.023 μL/mL, there was no repellency observed until 10 min, but 100% repellency was achieved after 25 min. One of the earlier studies on *R. chalepensis* essential oil revealed its insect-repellent property against mosquitoes, mainly *Mansonia* mosquito (family: Culicidae) populations (*Mansonia uniformis*, *M. nigerrima and M. africana*) in Gambella, Ethiopia [105]. At a concentration of 50% essential oil, the highest level of repellency showed 91.6% protection, while it was 78.0% when 40% concentration was applied. It can be mentioned here that the percentage protection method appears to be a preferred method for evaluating insect repellency to other conventional methods. Conti et al. [46] reported the mosquito-repellent activity of the essential oils of the aerial parts of *R. chalepensis* collected from El Ala, as well as grown in Tunisi, Tunisia, against the most invasive Asian tiger mosquito, *Aedes albopictus* (Diptera: Culicidae) using the human-bait technique. Essential oil obtained from the wild sample was found to be a good repellent against *Aedes albopictus;* the RD_50_ (repellent dose 50%) and RD_90_ (repellent dose 90%) were 0.000215 μL/cm^2^ and 0.007613 μL/cm^2^ of the skin, respectively. At the highest concentration (0.08 μL/cm^2^ of skin), this essential oil could repel 50% of mosquitoes for at least 45 min.

Biting deterrence and repellent properties of the essential oils of the aerial parts of *R. chalepensis* growing in Turkey were assessed against mosquitoes [47]. The major compounds present in this essential oil, e.g., 2-undecanone, 2-noanone, and 2-nonyl acetate, were also tested. The biting deterrent activity of the essential oil (10 and 50 μg/cm^2^), 2-undecanone (8.5 μg/cm^2^), 2-nonanone (9 μg/cm^2^), and 2-nonyl acetate (9.3 μg/cm^2^) was similar to the positive control *N*,*N*-diethyl-*meta*-toluamide (DEET) at 4.8 μg/cm^2^ against *Aedes aegypti*, and the activity of the essential oil at 50 μg/cm^2^ against another mosquito species, *Anopheles quadrimaculatus*. The cloth patch assay with the essential oil and 2-undecanone revealed their repellent activity against *Aedes aegypti* at 187 μg/cm^2^ and 108.9 μg/cm^2^. The essential oil obtained from the aerial parts of *R. chalepensis* from Italy showed significant oviposition deterrence activity against the mosquito *Aedes albopictus* (Diptera: Culicidae) [13], and the arm in cage tests established that this essential oil could repel females of *Aedes albopictus* with an ED_50_ (effective dose 50%) value of 0.2 nL/cm^2^ of skin.

*Ruta* essential oils, e.g., essential oil of *Ruta graveolens*, was previously tested for repellent efficacy against other mosquitoes, e.g., *A. aegypti* with a minimum effective dose of 0.187 mg/cm^2^ using the cloth patch assay [106]. Prior to this study, Soares et al. [107], examined the insect repellent activity of the essential oil from *R. graveolens* against the Cayenne tick *Amblyomma cajennense* (Acari: Ixodidae) nymphs, and found this essential oil to have little or no repellent effect.

The published reports as described above on the insect-repellent activities of *Ruta* essential oils, especially against various mosquito species, suggest that *Ruta* essential oils could be commercially exploited for mosquito-repellent product development.

### 3.9. Larvicidal Activity

*Ruta* essential oils possess larvicidal properties. A dose-dependent larvicidal effect of the essential oil of *R. montana* was observed against mosquito larvae; even a dose as low as 1.20 × 10^−3^% could exert an excellent outcome with 100% death of the larvae after three days [68]. The length required for achieving 100% mortality could be reduced significantly from 3 days down to 1 h by increasing the concentration of the applied essential oil (9.6 × 10^−3^%). Akkari et al. [38] studied the insecticidal property of the essential oils obtained from flowers and leaves of *R. chalepensis* using a larvicidal assay involving the larvae of *Orgyia trigotephras*, where mean mortality time of the larvae subjected to the flower was higher than those treated with the essential oil of the leaves. The larvicidal activity of the essential oil of *R. graveolens* was demonstrated against the fourth instar larvae of *Culiseta longiareolata* mosquitoes [108], where a concentration-dependent activity was observed. The LC_25_ (lethal concentration 25%) and LC_50_ (lethal concentration 50%) were determined as 6.96 and 10.11 ppm, respectively.

The larvicidal activity of *R. chalepensis* essential oil was reported against mosquito larvae about two decades ago [109]. Conti et al. [46] reported larvicidal activity of the essential oils of the aerial parts of *R. chalepensis* collected wild from El Ala, as well as grown in Tunisi, Tunisia, against the fourth instar larvae of the most invasive Asian tiger mosquito, *Aedes albopictus* (Diptera: Culicidae). Only a slight difference in the activities between the essential oils from the cultivated sample and the wild grown sample was observed, as is evident from the LD_50_ values of 33.2% and 35.7%, respectively, in the larvicidal assay.

In another similar work, the larvicidal activity of the essential oils of the aerial parts of *R. chalepensis* growing in Turkey and its major components were assessed against *Aedes aegypti* and *Anopheles quadrimaculatus* mosquito larvae [47]. The larvicidal activities of the essential oil against *Aedes aegypti* and *Anopheles quadrimaculatus* were evident from the LD_50_ values, 22.2 ppm and 14.9 ppm at 24 h, respectively. One of the major compounds of this essential oil, 2-undecanone, showed more prominent larvicidal activity that the essential oil against *Aedes aegypti* (LD_50_ 14.37 ppm), while the activity of the other major component, 2-nonanone, was less potent (LD_50_ 106.9 ppm) than that of the essential oil. Furthermore, 2-nonyl acetate did not show any larvicidal activity at the tested concentrations. The LD_50_ values of 2-undecanone and 2-noanone against *Anopheles quadrimaculatus* were 14.2 ppm (similar to the essential oil) and 65.5 ppm, respectively, at 24 h. Similar larvicidal activity of the essential oil of *R. chalepensis* was reported against the larvae of *Aedes aegypti*, the New Orleans strain of mosquitoes [30], where the LD_50_ value was determined as 2.69 μg/mL at 24 h, while this value was 20.13 μg/mL at 24 h against the larvae of the local population of *Aedes aegypti* found in Nuevo Leon, Mexico. The essential oil obtained from the aerial parts of *R. chalepensis* from Italy showed significant larvicidal activity against the mosquito *Aedes albopictus* (Diptera: Culicidae) [13], and the LD_50_ was determined as 93.6 μL/L.

It is not exactly a larvicidal activity, but the larvae-repellent activity of the essential oil of *R. graveolens* against the neonate larvae of the codling moth (Lepidoptera: Tortricidae) using the barrier test was reported [110]. The repellent property was indicated by a significant number of turn-arounds by codling moth larvae at the barrier. Time required for neonate codling moth larvae to cross essential oil applied to a glass rod in a laboratory was 2.5 min at a concentration of 0.1 mg/mL of *R. graveolens* essential oil, and the time increased as the concentration increased, e.g., the time was 42.3 min at a concentration of 100 mg/mL. The arrestant property of this essential oil was also tested against these larvae, but no detectable activity was observed. da Silva et al. [51] reported the larvicidal activity of the essential oil of fresh leaves of this *Ruta* species, grown in Brazil, against the third instar larvae of *Aedes aegypti* revealing the LD_50_ and LD_90_ values of 39.6% and 64.2%, respectively. The essential oil of *R. graveolens*, obtained by microwave hydrodistillation, was found to possess the highest larvicidal activity among several Colombian plants tested for this activity against third instar larvae of *Culex quinquefasciatus*, with the LD_50_ value of 7.2 μg/mL [53].

There are only a handful of reports available on the larvicidal activity of the essential oil from *R. montana*. The essential oil of *R. montana* was assessed for the larvae-repellent and larvicidal activities against the flour moth (*Ephestia kuehniella*) larvae [111]. Interestingly, instead of larvae-repellent, this essential oil demonstrated larvae-attractant activity. However, this essential oil showed larvicidal activity with the larval mortality rate of 56.7% during the first 24 h, and an LD_50_ value of 11.6 μL/L.

The promising published reports as described above on the larvicidal activities of *Ruta* essential oils, especially against various mosquito species, suggest that *Ruta* essential oils could potentially be exploited commercially as mosquito control agents.

### 3.10. Nematocidal and Anthelminitic Activity

Nematodes are parasitic worms of different types that live in animals, humans, insects, and plants. Different plant extracts as well as plant essential oils have long been used in traditional medicines as nematocidal and anthelmintic agents [112]. *Ruta* essential oils have been studied for nematocidal or anthelmintic property. From an ethnopharmacological survey, it is obvious that extracts and essential oils of *Ruta* species, particularly *R. graveolens*, have long been used as an anthelmintic agent in traditional medicine in Italy [97].

The nematocidal activity of the essential oil of fresh leaves of *R. graveolens*, growing in Brazil, against juvenile nematodes of *Meloidogyne incognita*, was evident from its LD_50_ and LD_90_ values of 267.2 and 482.0 ppm, respectively. A significant level of hatching inhibition activity, with effective concentration 50% (EC_50_) < 0.15 μL/mL of the essential oil of this *Ruta* species was reported against the Colombian root-knot nematode *Meloidogyne chitwoodi*, which is one of the major causes of damage in potato and tomato crops [113]. Earlier, the essential oil of *R. graveolens* from Portugal was shown to be active against the nematode *Bursaphelenchus xylophilus*, which is the causative nematode for the pine wilt disease that is common in Portuguese pine forests [114,115]. In the direct contact assay, this essential oil demonstrated nematocidal activity with an LC_100_ (lethal concentration 100%) value of <0.4 μL/mL [114].

A study, conducted by da Silva et al. [51], also established the nematocidal property of the essential oil of *R. graveolens*, and the activity was observed against the juvenile nematodes of *Meloidogyne incognita* with IC_50_ (inhibition concentration 50%) and IC_90_ (inhibition concentration 90%) values of 267.2 ppm and 482.0 ppm, respectively. The nematocidal activity could be attributed to the presence of aliphatic ketones, e.g., 2-undecanone, and 2-nonanone as the major compounds in this essential oil. A similar finding was also published for the essential oil of *R. graveolens* against *Meloidogyne incognita*, which was part of the search for plant essential oil-based biofumigants for the control of the root knot nematodes on tomato [116]. This essential oil was found to significantly reduce nematode multiplication and gall formation on tomato roots at a dose of 50 μL per kilogram of soil. In the most recent study [58], the essential oil of *R. graveolens* was shown to possess significant nematocidal property against the eggs and juveniles of the root-knot nematode *Meloidogyne incognita.* In the juvenile mortality assay, the mortality ranged from 31–56% after 24 h immersion in 0.78–6.25 μg/mL solutions of this essential oil, but the mortality rose above 90% after 8 h of exposure to a 1 μg/mL concentration. In the egg hatchability bioassay, it was found that the essential oil of *R. graveolens* could significantly affect the hatchability of *M. incognita* eggs, bringing it down to only 1.2% after 96 h egg mass exposure to a 500 μg/mL solution.

Aliphatic ketones from the *R. chalepensis* essential oil were found to induce paralysis on root knot nematodes, which are well-known as plant-parasitic nematodes from the genus *Meloidogyne* [25]. The nematocidal activity of this essential oil against second-stage juveniles of *Meloidogyne incognita* and *M. javanica* was evident from the EC_50_ (effective concentration 50%) values of 77.5 and 107.3 μg/mL, respectively, after one day of treatment. The major aliphatic ketone, 2-undecanone, present in this essential oil was also tested for nematocidal activity against these nematodes, and the EC_50_ values were determined as 206 and 22.5 μg/mL, respectively. In addition to 2-undecanone, among the other active components, 2-dodecanone and 2-decanone were the most active, followed by 2-nonanone and 2-tridecanone.

Akkari et al. [38] reported the nematocidal activity of the essential oils of flowers and leaves of *R. chalepensis* against the gastrointestinal parasitic nematode *Haemonchus contortus* using an in vitro nematocidal assay; the activity was evident from the inhibitory effect of these oils on egg hatching and worm motility as compared to those of the positive control, albendazole. At tested concentrations (0.125–1.0 mg/mL), essential oils form the flowers and leaves exhibited ovicidal activity, and the essential oil form the leaves was more potent than that of the flowers with IC_50_ values of 0.145 and 0.398 mg/mL, respectively. The highest level of inhibition of motility (87.5%) was achieved with the concentration of 1 mg/mL and the exposure time of 8 h for the essential oil of the leaves, whereas at the same concentration and exposure time, the inhibition of motility was found to be 75% with the essential oil of the flowers.

A dose-dependent nematocidal activity of the essential oil of the aerial parts of *R. chalepensis* as well as its major compounds, e.g., 2-decanone, 2-nonanone, and 2-undecanone, was assessed against ewe gastrointestinal nematodes, e.g., *Haemonchus contortus*, *Teladorsagia* sp., and *Trichostrongylus* sp. [12]. The EC_50_ value after one day of exposure to this essential oil was 1.29 mg/mL, and that of 2-decanone, 2-nonanone, and 2-undecanone were 0.07, 0.25, and 0.88 mg/mL, respectively, revealing that 2-decanone was the most potent nematicide among the tested compounds.

### 3.11. Miscellaneous Activity

Apart from the activities discussed above, there are a few other activities of *Ruta* essential oils reported in the literature. Drioueche et al. [117] unveiled the possibility for the use of the essential oil from *R. montana*, which contains high amounts of aliphatic ketones of different bond lengths (Table 1), as a starting material for the synthesis of a natural emulsifier. Although not exactly a bioactivity, the steel corrosion inhibitory activity of the essential oil of *R. chalepensis* was evaluated in aqueous hydrochloric acid (HCl) solution (1 M) using the weight-loss method, potentiodynamic polarization, and electrochemical and impedance spectroscopy, and an increase in efficiency of 77% was found at a concentration of 2.5 mL/L [19].

## 4. *Ruta* Essential Oils, Nanotechnology, and Chitosan

The term ‘nanotechnology’ was first introduced by Norio Taniguchi in 1974 [118,119]. However, the pioneering concept of nanotechnology was first presented by Richard Feynman in 1959. Nanotechnology can be defined as the manipulation of matter on an atomic, molecular, and supramolecular scale. It utilizes particles with dimensions of 1–100 nanometers (nm), which are referred to as nanoparticles. This technology has already been playing a major role in advancing and impacting almost all areas of science, especially, drug formulation and delivery. Nanotechnology has recently been applied to the targeted delivery of essential oils in order to enhance their efficacies, and publications demonstrating this new approach have just begun to appear in the literature; for example, enhanced efficacy of essential oils has been demonstrated with chitosan nanoparticles loaded with essential oils against multi-drug-resistant (MDR) *Klebsiella pneumoniae* [120], and other bacterial species, e.g., *Enterococcus faecalis*, *Escherichia coli*, *Staphylococcus aureus* and *Pseudomonas aeruginosa* [121]. Because of the nonpolar nature of the essential oils, microencapsulation and/or nanoencapsulation often enhances delivery efficiency of essential oils. It can be noted that chitosan is a biopolymeric sugar (polysaccharide) molecule found in the hard outer shell of shellfish, e.g., crab, lobster and shrimp, and chitosan nanoparticles are used in the delivery of drugs and other bioactive molecules. The hydrophilic nature of chitosan is a weak barrier, but this drawback can be mitigated by creating nanocomposites incorporating hydrophobic essential oils.

Anthracnosis, caused by the pathogenic fungi *Colletotrichum gloeosporioides*, is a common problem for preserving guava fruits, which are generally highly perishable. Tova et al. [122] proposed an emulsion coating of chitosan with *R. graveolens* essential oil (undecanone 42.6% and 2-nonanone 23.5%) as a preventative measure for minimizing the loss of guava fruits (*Psidium guajava* L.) and extending their storage/preservation time at room temperature. While in this emulsion, the essential oil, as well as chitosan, was the active antifungal agent. Additionally, chitosan helped with the emulsion stability. It is known that the smaller the particle size, the larger is the surface area. Thus, it could be envisaged that the reduction of the particle size by using chitosan nanoparticles would further enhance the quality of this emulsion. Most recently, the protecting effect of chitosan-*Ruta graveolens* essential oil emulsion against *Colletotrichum gloeosporioides* has also been shown in papaya fruits (*Carica papaya* L.) [123].

In a similar study, Gonzalez-Locarno et al. [124] demonstrated that an edible coating, based on chitosan-*Ruta graveolens* essential oil, could prevent microbial decay of gooseberries (*Physalis peruviana* L.) caused by molds and yeasts, and thus extend storage length, and the chitosan-*Ruta graveolens* essential oil-based coating was found to be useful for increasing the shelf-life of tomatoes (*Solanum lycopersicum* L.) at low temperature storage by inhibiting the growth of bacteria, mold, and yeast [125].

## 5. Conclusions

*Ruta chalepensis*, *R. graveolens*, and *R. montana* are three most extensively studied *Ruta* species for the composition and bioactivities of their essential oils, and most of the *Ruta* samples studied were from Algeria and Tunisia. The essential oils of *Ruta* are dominated by two aliphatic ketones, 2-undecanone and 2-nonanone, but considerable variations in their amounts could be observed, which is mainly due to differences in climatic conditions, soil quality, geographical sources, collection time, and processing methods. While various bioactivities of *Ruta* essential oils have been documented in the literature, significant variations in bioactivity profiles and potencies do exist among essential oils from *Ruta* samples. Most of the bioactivities reported in the literature involve preliminary in vitro screening, and probably have limited clinical relevance. Moreover, it is worth mentioning that there is not enough information available on definitive correlations between biological activities and the compositions of these essential oils, leaving several unresolved questions on the efficacy and practical applications of these essential oils. The true potential of *Ruta* essential oils resides in their non-medicinal bioactivities, e.g., pesticidal, larvicidal, insecticidal and insect-repellent properties, which can be exploited further for development of commercial agrochemical products.

## Figures and Tables

**Table 1:** Major components of the essential oils of *Ruta* species from different geographical origins.

*Ruta* Species	Geographical Sources (Region)	Plant Parts (Yield)	Major Components	References
*Ruta angustifolia* Pers.	Algeria (Tlemcen)	Air-dried aerial parts (1.49%)	2-Undecanone (82.5%) and 2-decanone (10.0%)	[18]
*Ruta chalepensis* L.	Algeria (Ain-delfa)	Air-dried aerial parts (0.90%)	2-Undecanone (67.0%), 2-decanone (9.0%), 6-(3′,5′-benzodioxyl)-2-hexanone (6.3%), and 2-dodecanone (4.0%)	[19]
	Algeria (Blida)	Air-dried aerial parts (0.40%)	2-Undecanone (35.5%), 2-methyl-1-decanol (8.6%), and 2-dodecanone (6.9%)	[20]
	Chile	Air- and oven-dried (20 °C) aerial parts (0.30%)	2-Nonanone (41.7%) and 2-undecanone (40.1%)	[21]
	Colombia (Cacota)	Shade-dried leaves (0.12%)	2-Undecanone (39.4%), 2-nonanone (37.1%), 2-decanone (2.8%), and nonyl acetate (2.2%)	[22]
	Cuba	Air-dried aerial parts (0.30%)	2-Undecanone (34.9%) and 2-nonanone (25.2%)	[23]
	Greece	Air-dried aerial parts (1.10%)	2-Methyloctyl acetate (44.0%), β-phellandrene (10.8%), 2-nonanol (7.2%), and β-pinene (6.4%)	[24]
	Italy (San Alessio)	Fresh flowers (0.89%)	2-Nonanone (44.9%), 2-undecanone (44.9%), and limonene (1.8%)	[11]
		Fresh fruits (1.40%)	2-Nonanone (51.8%), and 2-undecanone (41.9%)	[11]
		Fresh leaves (1.10%)	2-Nonanone (69.9%), 2-undecanone (15.5%), and limonene (3.1%)	[11]
		Fresh stems (0.97%)	2-Nonanone (51.3%), 2-undecanone (33.0%), and limonene (3.7%)	[11]
	Italy (Cagliari)	Air-dried aerial parts (0.36%)	2-Undecanone (~56.0%), 2-nonanone (~36.0%), 2-decaone (~2.5%), and 2-nonanol (~2.0%)	[25]
	Italy (Cagliari)	Air-dried aerial parts (1.09%)	2-nonanone (25.3%), 2-undecanone (24.0%), limonene (12.8%), octyl acetate (10.4%), geijerene (5.7%), and 2-decaone (3.7%)	[12]
	Italy (Monte Pisano)	Air-dried aerial parts (0.79%)	2-Nonanone (56.7%), 2-nonanol (3.5%), and limonene (2.2%)	[13]
	India	Fresh flowers (0.69%)	2-Undecanone (67.8%), 2-nonanone (10.3%), 2-decanone (3.1%), and 2-nonyl acetate (2.8%)	[26]
		Fresh fruits (0.88%)	2-Undecanone (60.0%), 2-dodecanone (11.6%), 2-nonanone (5.2%), and 2-nonyl acetate (4.4%)	[26]
		Fresh leaves (0.56%)	2-Undecanone (41.3–47.7%), 2-nonanone (13.8–33.6%), 2-nonyl acetate (9.0–15.3%), 2-decanone (2.6–3.1%), and 2-dodecanone (0.7–2.2%)	[26]
	Iran	Air-dried aerial parts (1.3%)	2-Undecanone (52.5%), 2-nonanone (24.1%), and nonyl acetate (9.1%)	[27]
	Jordan	Air-dried (in the dark) aerial parts (0.83%)	2-Cyclohexen-1-one,3-[(2,3,4,9 tetrahydro-1H-pyrido[3,4 b]indole-1-yl) methyl] (45.9%) and 2-nonanone (19.5%)	[28]
	Lebanon	Fresh leaves and stems (0.12%)	2-Nonanone (42.5%), 2-undecaone (41.4%), and terpen-4-ol (2.2%)	[29]
		Fresh leaves (0.12%)	2-Nonanone (51.7%) and 2-undecaone (36.7%)	[29]
	Mexico	Fresh aerial parts	2-Undecaone (43.7%) and 2-nonanone (35.4%)	[30]
	Morocco (Central plateau)	Shed-dried aerial parts (2.72%)	2-Undecanone (64.4%), piperonyl piperazine (11.9%), 2-decanone (5.1%), and 2-dodecanone (4.5%)	[31]
	Morocco (High Atlas Mountains)	Air-dried leaves (0.56%)	2-Undecaone (49.1%), 2-nonanone (33.2%), limonene (4.2%), and 2-decanone (2.7%)	[10]
	Morocco	Air-dried aerial parts (0.66%)	2-Undecaone (93.1%)	[32]
	Palestine (Jerusalem, Hebron and Jenin)	Shade-dried leaves (0.23%)	2-Undecanone (7.7–44.3%) and 2-nonanone (8.2–43.0%)	[33]
	Peru	Fresh aerial parts (0.22%)	2-Undecanone (58.2%) and 2-nonanone (25.3%)	[14]
	Poland	Shade-dried leaves (0.26%)	2-Undecanone (50.0%), 2-nonanone (35.0%), and geijerene (9.2%)	[17]
		Shade-dried roots (0.19%)	Octyl acetate (29.0%), methyl decanoate (22.1%), and phytyl acetate (17.2%)	[17]
		Shade-dried stems (0.82%)	2-Decanone (21.2%), geijerene (19.2%), 2-nonanone (16.1%), and 2-undecaone (9.1%)	[17]
	Portugal (Madeira island)	Fresh leaves (0.32%)	2-Undecanone (53.0%), (*E*)-2-octenal (28.0%), and 2-nonanone (10.0%)	[34]
	Spain	Shade-dried aerial parts (0.43%)	2-Undecanone (64.9%)	[35]
	Tunisia (Sdira and Thoujene)	Shade-dried fruits (3.0%)	2-Undecanone (57.5 and 58.4%), 2-nonanone (19.0 and 23.3%), and octyl acetate (3.4 and 5.1%)	[36]
		Shade-dried leaves (1.40%)	2-Undecanone (23.0 and 27.9%), 2-nonanone (21.1 and 16.7%), octyl acetate (26.6 and 26.8%), and chalepensin (3.0 and 2.3%)	[36]
		Shade-dried stems (0.31%)	2-Undecanone (31.9 and 37.7%), 2-nonanone (23.0 and 22.1%), octyl acetate (11.0 and 12.1%), and decyl acetate (4.2 and 5.4%)	[36]
	Tunisia (El Fahs region)	Shade-dried aerial parts (0.30%)	2-Undecanone (87.2%)	[37]
	Tunisia (El Fahs)	Fresh flowers (1.75%)	2-Undecanone (89.9%) and 2-decanone (4.2%)	[38]
		Fresh leaves (0.69%)	2-Undecanone (85.9%), 2-decanone (5.6%), and piperazine (3.0%)	[38]
	Tunisia (Thoujene)	Shade-dried fruits (2.5%)	2-Undecanone (58.4%), 2-nonanone (19.1%), and 2-undecanol (4.1%)	[39]
		Shade-dried leaves (0.90%)	2-Undecanone (27.9%), 2-nonanone (16.7%), octyl acetate (26.8%), decyl acetate (9.4%), and 2-nonanol (3.8%)	[39]
		Shade-dried stems (0.30%)	2-Undecanone (37.7 %), 2-nonanone (22.1%), octyl acetate (12.1%), decyl acetate (5.4%), and 2-undecanol (2.9%)	[39]
	Tunisia (Beja)	Air-dried leaves (0.90%)	Menthol (43.9%), linalool (42.1%), and 2-hexanal (5.8%)	[9]
	Tunisia	Air-dried aerial parts at flowering and post-flowering stages (0.70 and 0.50%, respectively))	2-Undecanone (33.4–49.8%), 2-heptanol acetate (13.5–15.4%), and α-pinene (9.8–11.9%)	[40]
	Tunisia (El Hamma)	Shade-dried leaves (0.66%)	2-Undeconone (48.6%), 1-nonene (18.4%), and 2-nonanone (3.5%)	[41]
		Share-dried stems (0.66%)	2-Undeconone (50.6%), 1-nonene (10.9%), 2-nonanone (5.4%) and 1-dodecene (3.7%)	[41]
	Tunisia (Kef)	Air-dried leaves (0.85%)	Menthol (49.9%), linalool (31.1%), and 2-hexanal (5.2%)	[42]
	Tunisia (El Fahs)	Shade-dried flowers (0.77%)	2-Undecanone (100%)	[43]
		Shade-dried leaves (0.55%)	2-Undecanone (69.2%), camphor (2.5%), 2-decanone (2.4%) and 2-dodecanone (2.0%)	[43]
		Fresh leaves (0.98%)	2-Undecanone (77.2%), 2-decanone (9.0%), and 2-dodecanone (2.4%)	[43]
		Shade-dried stems (0.70%)	Pulegone (32.1%)	[43]
	Tunisia (Mountain Traza)	Air-dried flowers (0.70–0.99%)	2-Undecanone (44.1–60.5%), 2-nonanol (21.5–36.4%), and 2-dodecanone (0.1–2.8%)	[44]
		Air-dried fruits (1.73%)	2-Undecanone (0.17–41.0%), 2-nonanol (0.86–11.6%), and 2-dodecanone (5.8–80.0%)	[44]
		Air-dried leaves (0.39–0.57%)	2-Undecanone (33.5–36.6%), 2-nonanol (28.1–45.1%), and 2-dodecanone (0.1–0.2%)	[44]
		Air-dried stems (0.49–0.66%)	2-Undecanone (26.7–33.5%), 2-nonanol (28.1–40.5%), and 2-dodecanone (0.2–13.6%)	[44]
	Tunisia (Bouaouene)	Fresh aerial parts (0.39%)	2-Undecanone (51.2%), 2-nonanone (39.2%), and 2-decanone (2.3%)	[45]
	Tunisia (El Ala)	Air-dried aerial parts of wild plants (0.56%)	2-Nonanone (37.4%), 2-undecanone (20.5%), and 2-methyl-octyl acetate (19.0%)	[46]
	Tunisia (Tunisi)	Air-dried parts of cultivated plants (0.60%)	2-Undecanone (39.3%), 2-nonanone (20.5%), and 2-methyl-octyl acetate (7.6%)	[46]
	Turkey	Fresh aerial parts (1.10%)	2-Undecanone (43.2%), 2-nonanone (27.9%), and 2-nonyl acetate (10.6%)	[47]
*Ruta chalepensis* subsp. *angustifolia* (Pers.) P. Cout.	Algeria	Shade-dried aerial parts (0.91%)	2-Undecanone (84.7%)	[15]
	Algeria (Jijel)	Fresh aerial parts (0.80%)	2-Undecanone (83.4%), carvacrol (4.1%), and 2-nonanone (4.0%)	[48]
	Algeria (Boudouaou)	Air-dried aerial parts (0.27%)	2-Undecanone (28.2%), 2-nonanone (20.0%), 2-methyloctyl acetate (12.7%), and 2-methyldecyl acetate (5.8%)	[49]
*Ruta chalepensis* var. *bracteosa* (DC) Boiss.	Algeria (Ain Temouchent)	Air-dried aerial parts (0.90%)	2-Nonanone (32.8%), 2-undecanone (32.6%), 1-nonene (14.0%), α-limonene (5.3%), and 2-decanone (2.4%)	[18]
*Ruta chalepensis* subsp. *latifolia* (Salisb.) Linds.	Algeria	Shade-dried aerial parts (0.69%)	2-Undecanone (51.2%), 2-nonanone (20.1%), 2-octyl-methyl acetate (15.1%), and 2-dectyl acetate (3.3%)	[15]
*Ruta graveolens* L.	Algeria (Anaba)	Air-dried aerial parts (0.18%)	2-Undecanone (55.4%), 2-nonanone (21.6%), 1-nonene (4.4%), and α-limonene (4.3%)	[18]
	Brazil (Maranhão)	Fresh leaves (1.29%)	2-Undecanone (47.2%), 2-nonanone (39.2%), octyl acetate (7.3%), and 2-decanone (2.0%)	[50]
	Brazil (Ceara)	Fresh leaves (0.10–0.90%)	2-Undecanone (37.0–58.2%) and 2-nonanone (17.6–53.1%)	[51]
	Bulgaria	Shade-dried fruits (0.12%)	2-Nonanone (60.1%), benzaldehyde (7.4%), and 2-undecanone (7.0%)	[52]
	China	Air-dried aerial parts (0.99%)	2-Undecanol acetate (19.2%) and 2-undecanol 2-methylbutyl ester (8.9%)	[8]
	Colombia (Santander)	Shade-dried aerial parts (1.60%)	2-Nonanone (35.4%), 2-undecanone (30.5%), and 2-decanone (3.4%)	[53]
	Egypt	Air-dried leaves (0.34%)	2-Undecanone (62.0%) and 2-nonanone (18.0%)	[54]
	Egypt (Minia)	Fresh flowers (0.215%)	2-Undecanone (70.2–84.6%) and 2-noanone (3.0–7.4%)	[55]
		Fresh leaves (0.37%)	2-Undecanone (52.5–58.1%) and 2-noanone (18.0–25.1%)	[55]
	India (Rayalaseema region)	Air-dried aerial parts (1.29%)	2-Undecaone (43.7%), 2-nonanone (16.1%), 2-tridecanone (2.6%), and 2-decanone (2.6%)	[56]
	India (Orissa)	Fresh leaves (field grown, 0.80%)	2-Undecanone (45.4%), 2-nonanone (21.4%), 2-nonyl acetate (6.8%), and 2-dodecanone (4.1%)	[57]
		Fresh leaves (micropropagated, 0.84%)	2-Undecanone (48.1%), 2-nonanone (22.5%), 2-nonyl acetate (6.8%), and 2-dodecanone (4.0%)	[57]
	Italy	Commercial oil	2-Undecanone (83.2%) and carvacrol (15.0%)	[58]
	Korea	Air-dried aerial parts (0.06%)	2-Isopropyl-5-methylphenol (31%), Pentadecanol (18.5%), 1-methyltridecyl pentanoate (12.1%), 4-hexadecanyl pivalate (6.1%), and 2-acetoxytridecane (5.6%)	[59]
	Peru	Fresh aerial parts (0.27%)	2-Undecanone (40.9%), 2-nonanone (29.0%), and β-caryophyllene (3.4%)	[14]
	Portugal	Air-dried aerial parts (0.81%)	2-Undecanone (91.0%) and 8-phenyl-2-octanone (7.0%)	[60]
	Russia	Genetically transformed fresh roots (0.23%)	Geijerene (67.0%)	[16]
	Tunisia (Tozeur oases)	Air-dried leaves (0.21%)	1-Nonene (19.4%), 2-undecanone (16.2%), and 2-nonanone (11.9%)	[61]
	Tunisia (Tunis)	Fresh aerial parts (1.67%)	2-Undecanone (56.9%), 2-nonanone (23.6%), and 1-nonen (4.4%)	[62]
	Tunisia (Bizerta)	Oven-dried (40–60 °C) flowers (0.25–0.43%)	2-Undecanone (22.0–31.0%), 2-nonanone (10.5–12.3%), 2-dodecanone (9.8–12.3%), 2-tridecanone (10.3–11.7%), and limonene (5.4–8.3%)	[63]
		Oven-dried (40–60 °C) leaves (0.57–0.78%)	2-Undecanone (26.8–37.3%), 2-nonanone (10.5–12.8%), 2-dodecanone (3.2–5.7%), 2-tridecanone (5.1–5.3%), and limonene (2.6–5.1%)	[63]
		Oven-dried (40–60 °C) stems (0.42–0.63%)	α-Eudesmol (48.8–58.5%) and octanoic acid (28.1–30.5%)	[63]
	Tunisia (Jemmel)	Fresh leaves (0.30%)	2-Nonanone (38.7%), 2-undecanone (27.3%), and 2-nonanol (12.3%)	[64]
		Fresh stems (0.10%)	2-Undecanone (40.3%), 2-nonanone (35.0%), and 2-nonanol (3.8%)	[64]
	Tunisia (Tunis)	Shade-dried aerial parts (0.77%)	2-Undecanone (60.6%), borneol (12.0%), 2-dodecanol (7.9%), 2-nonanone (4.9%), and 2-dodecanone (3.7%)	[65]
	Turkey	Flowers and leaves (1.25%)	2-Undecanone (64.8%) and 2-nonanone (13.8%)	[66]
*Ruta montana* L.	Algeria (Blida, Bouira, Boumerdes, Djelfa, M’sila, Tipaza and Tizi ouzou)	Shade-dried aerial parts (0.38–1.45%)	2-Undecanone (27.2–81.7%), 2-nonanone (1.9–39.5%), and 2-nonanyl acetate (trace-24.8%)	[67]
	Algeria	Shade-dried aerial parts (0.65%)	2-Undecanone (20.9–70.1%), *E*-caryophyllene (5.0–9.1%), and caryophyllene oxide (2.5–3.6%)	[15]
	Algeria (Tipaza)	Fresh aerial parts (0.97%)	2-Undecanone (67.0%), 2-decanone (9.0%), and 2-dodecanone (4.0%)	[68]
		Air-dried aerial parts (0.60%)	2-Undecanone (67.4%), 2-decanone (7.6%), and 2-dodecanone (4.0%)	[68]
	Algeria (Oran)	Air-dried aerial parts (1.63%)	2-Undecanone (32.8%), 2-nonanone (29.5%), nonanol-2-acetate (18.2%), and psoralen (3.5%)	[69]
	Algeria (Hammam Melouane)	Shade-dried aerial parts (1.80%)	2-Undecanone (71.4%), 2-tridecanone (10.5%), 2-dodecanone (8.1%), and 2-decanone (5.4%)	[70]
	Morocco (Boulemane region)	Air-dried aerial parts (1.46%)	2-Undecanone (82.6%), 2-undecanol (2.9%), and 2-undecanol acetate (2.1%)	[71]
	Morocco (Taza)	Air-dried aerial parts (0.37%)	2-Undecanone (64.9%), camphor (3.8%), and cyclopropane carboxylic acid (3.7%)	[72]
	Tunisia (Tunis)	Fresh aerial parts (1.21%)	2-Undecanone (88.8%) and 2-decanone (4.9%)	[62]
	Tunisia (Sfax)	Air-dried leaves (0.26%)	1-Butene (38.3%), 2-butene (22.6%, methylcyclopropane (15.5%), and caryophyllene oxide (8.2%)	[73]
	Tunisia (Joumine)	Air-dried leaves (0.66%)	2-Undeconone (52.2%), 1-nonene (13.5%), 2-nonanone (10.1%), and 2-undecanol (2.4%)	[41]
		Air-dried stems (0.66%)	2-Undeconone (44.9%), 1-nonene (5.8%) and 2-nonanone (3.9%)	[41]
*Ruta tuberculata* Forssk.	Algeria (Bechar)	Air-dried aerial parts (0.11%)	Piperitone (13.6%), *trans-p-*menth-2-en-1-ol (13.1%), *cis-*piperitol (12.3%), *cis-p-*menth-2-en-1-ol (13.1%), *trans-*piperitol (4.1%), and 2-undecanone (1.6%)	[18]

**Table 2:** Antibacterial activity of *Ruta* essential oils.

*Ruta* Essential Oil Source	Activity against Bacterial Species (Zones of Inhibition in mm and/or MIC in μg/mL)	References
*Ruta angustifolia* Pers. Aerial parts	No detectable activity against *Acinetobacter baumanii*, *Enterobacter cloacae*, *Escherichia coli*, *Klebsiella pneumoniae*, *Listeria monocytogenes*, *Proteus mirabilis*, *Pseudomonas aeruginosa*, *Salmonella typhi*, and *Staphylococcus aureus* Active against *Bacillus cereus* (10 mm), *Enterococcus faecalis* (8 mm), and *Citrobacter freundii* (7 mm)	[18]
*Ruta chalepensis* L. Aerial parts	Plant pathogenic bacterial species, *Clavibacter michiganensis* subsp. *michiganensis* and *Xanthomonas albilineans*	[23]
*Ruta chalepensis* L. Aerial parts	*Streptococcus suis*	[81]
*Ruta chalepensis* L. Aerial parts	*Bacillus subtilis* (24 mm), *Escherichia coli* (22 mm), *Klebsiella pneumoniae* (25 mm), and *Staphylococcus aureus* (24 mm)	[20]
*Ruta chalepensis* L. Leaves	No detectable activity against *Aeromonas hydrophila*, *Bacillus subtilis*, *Escherichia coli*, *Listeria monocytogenes*, *Pseudomonas aeruginosa*, *Salmonella typhimurium*, and *Staphylococcus aureus*	[9]
*Ruta chalepensis* L. Leaves	*Escherichia coli*, *Listeria monocytogenes*, and *Pseudomonas aeruginosa*	[17]
*Ruta chalepensis* L. Leaves	*Escherichia coli* (750 μg/mL), *Pseudomonas aeruginosa* (7000 μg/mL), *Staphylococcus aureus* (2500 μg/mL) and methicillin resistant *Staphylococcus aureus* (MRSA) (4000 μg/mL)	[33]
*Ruta chalepensis* L. Leaves	*Escherichia coli* (15.6 μg/mL), *Pseudomonas aeruginosa* (125 μg/mL) and *Staphylococcus aureus* 15.6 μg/mL)	[22]
*Ruta chalepensis* L. Leaves and stems	*Escherichia coli* (>512 μg/mL) and *Staphylococcus aureus* (>512 μg/mL)	[29]
*Ruta chalepensis* L. Roots	*Listeria monocytogenes* and *Pseudomonas aeruginosa*	[17]
*Ruta chalepensis* L. Stems	*Escherichia coli*, *Listeria monocytogenes*, *Pseudomonas aeruginosa*, *Salmonella typhi*, and *Staphylococcus aureus*	[17]
*Ruta chalepensis* L. *var. bracteosa* Aerial parts	No detectable activity against *Citrobacter freundii*, *Enterobacter cloacae*, *Klebsiella pneumoniae*, *Listeria monocytogenes*, and *Pseudomonas aeruginosa* Active against *Acinetobacter baumanii* (12 mm), *Bacillus cereus* (12 mm), *Enterococcus faecalis* (10 mm), *Citrobacter freundii* (7 mm), *Escherichia coli* (7 mm), *Proteus mirabilis* (10 mm), *Salmonella typhi* (15 mm), and *Staphylococcus aureus* (17 mm)	[18]
*Ruta graveolens* L. Aerial parts	*Escherichia coli* (7 mm), *Klebsiella pneumoniae* (no activity), *Pseudomonas aeruginosa* (12 mm), and *Staphylococcus aureus* (16 mm)	[62]
*Ruta graveolens* L. Aerial parts	No activity against *Pseudomonas aeruginosa*	[82]
*Ruta graveolens* L. Aerial parts	*Helicobacter pylori*	[83]
*Ruta graveolens* L. Aerial parts	*Legionella pneumophila* (MIC < 0.02–0.40 μg/mL)	[65]
*Ruta graveolens* L. Aerial parts	*Acinetobacter baumannii* (19 mm, MIC 1.22 μg/mL), *Bacillus cereus* (28 mm, MIC 1.0 μg/mL), *Citrobacter freundii* (16 mm, MIC 1.0 μg/mL), *Enterobacter aerogenes* (13 mm, MIC 72.0 μg/mL), *Enterobacter cloacae* (18 mm, MIC 0.89 μg/mL), *Enterococcus faecalis* (27 mm, MIC 1.0 μg/mL), *Escherichia coli* (18 mm, MIC 0.65 μg/mL), *Klebsiella pneumoniae* (18 mm, MIC 1.58 μg/mL), *Listeria monocytogenes* (18 mm, MIC 0.70 μg/mL), *Micrococcus flavus* (21 mm, MIC 1.48 μg/mL), *Micrococcus luteus* (19 mm, MIC 1.0 μg/mL), *Proteus mirabilis* (22 mm, MIC 0.76 μg/mL), *Pseudomonas aeruginosa* (15 mm, MIC 1.0 μg/mL), *Salmonella typhimurium* (13 mm, MIC 1.34 μg/mL), and *Staphylococcus aureus* (23 mm, MIC 1.0 μg/mL)	[56]
*Ruta graveolens* L. Aerial parts	Food-borne bacterial species, *Bacillus cereus* (15 mm, MIC 75 μg/mL), *Listeria monocytogenes* (13 mm, MIC 75 μg/mL), *Salmonella enterica* (22 mm, MIC 25 μg/mL), *Staphylococcus intermedius* (14 mm, MIC 25 μg/mL), *Shigella sonnei* (14 mm, MIC 25 μg/mL), and *Salmonella typhimurium* (15 mm, MIC 25 μg/mL)	[59]
*Ruta graveolens* L. Aerial parts	No detectable activity against *Acinetobacter baumanii*, *Citrobacter freundii*, *Enterobacter cloacae*, *Klebsiella pneumoniae*, *Listeria monocytogenes*, and *Pseudomonas aeruginosa* Active against *Bacillus cereus* (12 mm), *Enterococcus faecalis* (9 mm), *Escherichia coli* (7 mm), *Proteus mirabilis* (7 mm), *Salmonella typhi* (12 mm), and *Staphylococcus aureus* (12 mm)	[18]
*Ruta graveolens* L. Leaves	*Bacillus cereus* (26 mm, MIC 1.0 μg/mL), *Enterobacter aerogenes* (13 mm, MIC 1.4 μg/mL), *Escherichia coli* (18 mm, MIC 1 μg/mL), *Micrococcus* *flavus* (19 mm, MIC 0.75 μg/mL), *Micrococcus luteus* (17 mm, MIC 0.89 μg/mL), *Pseudomonas aeruginosa* (8.0 mm, MIC 75.0 μg/mL), *Salmonella typhi* (12 mm, MIC 1.0 μg/mL), and *Staphylococcus aureus* (22 mm, MIC 1.0 μg/mL)	[50]
*Ruta graveolens* L. Leaves	*Bacillus cereus* (210 μg/mL), *Dickeya solani* (420 μg/mL) *Escherichia coli* (100 μg/mL), *Listeria monocytogenes* (210 μg/mL), *Micrococcus* *flavus* (210 μg/mL), *Pectobacterium atrosepticum* (310 μg/mL), *Pectobacterium carotovorum* subsp. *carotovorum* (110 μg/mL), *Pseudomonas aeruginosa* (350 μg/mL), and *Staphylococcus aureus* (100 μg/mL)	[54]
*Ruta graveolens* L. Leaves	*Staphylococcus aureus* (10–20 mm)	[78]
*Ruta graveolens* L. Leaves and flowers	*Escherichia coli* (MIC 7.5–7.9 μg/mL), *Klebsiella pneumoniae* (MIC 4.5–5.2 μg/mL), *Pseudomonas aeruginosa* (MIC 5.8–6.3 μg/mL), and *Staphylococcus aureus* (MIC 3.5–3.9 μg/mL)	[55]
*Ruta montana* L. Aerial parts	*Escherichia coli* (9 mm), *Klebsiella pneumoniae* (no activity), *Pseudomonas aeruginosa* (21 mm), and *Staphylococcus aureus* (21 mm)	[62]
*Ruta montana* L. Aerial parts	Not active against *Escherichia coli* Active against *Staphylococcus aureus* (18 mm)	[70]
*Ruta montana* L. Aerial parts	*Bacillus subtilis* (10–15 mm), *Enterobacter faceium* (11–13 mm), *Escherichia coli* (10–14 mm), *Klebsiella pneumoniae* (10–13 mm), *Pseudomonas aeruginosa* (9–13 mm), and *Staphylococcus aureus* (12–16 mm)	[67]
*Ruta montana* L. Aerial parts	*Bacillus subtilis* (21 mm, MIC 6250 μg/mL), *Escherichia coli* (not active), *Listeria innocua* (10 mm), and *Proteus mirabilis* (17 mm, MIC 6250 μg/mL), *Pseudomonas aeruginosa* (9 mm), and *Staphylococcus aureus* (12 mm, MIC > 25000 μg/mL)	[72]
*Ruta montana* L. Aerial parts	Activity against *Citrobacter koseri* (8 mm), *Corynebacterium* sp. (11 mm), *Enterococcus faecalis* (7 mm), *Enterococcus faecium* (17 mm), *Escherichia coli* (8 mm), *Klebsiella oxytoca* (8 mm), *Listeria* sp. (11 mm), *Proteus mirabilis* (7 mm), *Pseudomonas aeruginosa* (8 mm), *Salmonella* sp. (11 mm), *Serratia marcescens* (8 mm), *Staphylococcus aureus* (8 mm), *Staphylococcus haemolyticus* (8 mm), *Streptococcus acidominimus* (7 mm), *Streptococcus porcinus* (8 mm), and *Yersinia enterolitica* (8 mm). No activity against *Enterobacter aerogens*, *Enterobacter cloacae*, *Klebsiella pneumonie ssp. Pneumonie*, *Pseudomonas fluorescence*, *Pseudomonas putida*, *Shigella* sp., *Staphylococcus epidermidis*, *Streptococcus agalactiae*, and *Streptococcus groupe*	[71]
*Ruta montana* L. Aerial parts	*Agrobacterium tumefaciens*	[73]
*Ruta tuberculata* Forssk. Aerial parts	No detectable activity against *Acinetobacter baumanii*, *Citrobacter freundii*, *Enterobacter cloacae*, *Escherichia coli*, *Klebsiella pneumoniae*, *Listeria monocytogenes*, *Proteus mirabilis*, and *Pseudomonas aeruginosa.* Active against *Bacillus cereus* (12 mm), *Enterococcus faecalis* (14 mm), *Salmonella typhi* (8 mm), and *Staphylococcus aureus* (10 mm)	[18]

**Table 3:** Antifungal activity of *Ruta* essential oils.

*Ruta* Essential Oil Source	Activity against Fungal Species (Zones of Inhibition in mm or MIC in μg/mL)	References
*Ruta angustifolia* Pers. Aerial parts	*Alternaria alternaria* (25 mm), *Aspergillus flavus* (25 mm), *Aspergillus fumigatus* (20 mm), *Candida albicans* (35 mm), *Cladosporium herbarum* (18 mm), and *Fusarium oxysporum* (20 mm)	[18]
*Ruta chalepensis* L. *var. bracteosa* Aerial parts	*Alternaria alternaria* (10 mm), *Aspergillus flavus* (16 mm), *Aspergillus fumigatus* (23 mm), *Candida albicans* (15 mm), *Cladosporium herbarum* (35 mm), and *Fusarium oxysporum* (8 mm)	[18]
*Ruta chalepensis* L. Aerial parts	*Candida albicans* (28 mm) and *Saccharomyces cerevisiae* (27 mm)	[20]
*Ruta chalepensis* L. Aerial parts	*Alternaria* sp. (24 mm) and *Microdochium nivale* (29 mm)	[40]
*Ruta chalepensis* L. Aerial parts	*Alternaria solani* (22 mm)	[86]
*Ruta chalepensis* L. Leaves	*Candida albicans* (MIC 2750 μg/mL)	[33]
*Ruta chalepensis* L. Leaves	*Aspergillus flavus* (17 mm), *Aspergillus niger* (15 mm), and *Candida albicans* (11 mm)	[42]
*Ruta chalepensis* L. Leaves	Growth inhibition (100%) of *Fusarium culmorum*, *Fusarium graminearum*, *Fusarium polyphialidicum*, *Fusarium proliferatum*, and *Fusarium pseudograminearum* at a concentration of the essential oil at 20 μL/mL	[10]
*Ruta chalepensis* L. Leaves and stems	*Candida albicans* (512 μg/mL) and *Trichophyton rubrum* (512 μg/mL)	[29]
*Ruta chalepensis* L. Leaves, roots and stems	*Aspergillus* spp. (26 mm), *Saccharomyces cerevisiae* (26 mm), *Streptomyces griseus* (20 mm), *Fusarium solani* (19 mm), and *Penicillium thomii* (16 mm)	[17]
*Ruta graveolens* L. Aerial parts	*Aspergillus parasiticus* (28 mm)	[87]
*Ruta graveolens* L. Aerial parts	*Alternaria alternata* (22 mm), *Aspergillus flavus* (26 mm), *Aspergillus fumigatus* (14 mm), *Candida albicans* (35 mm), *Cladosporium herbarum* (26 mm), and *Fusarium oxysporium* (20 mm)	[56]
*Ruta graveolens* L. Aerial parts	*Melassezia furfur* (30 mm)	[88]
*Ruta graveolens* L. Aerial parts	*Candida albicans* (8.2 μg/mL), *Candida parapsilopsis* (16.4 μg/mL), *Candida glabrata* (4.1 μg/mL), and *Candida tropicalis* (131.0 μg/mL)	[89]
*Ruta graveolens* L. Aerial parts	*Alternaria alternaria* (25 mm), *Aspergillus flavus* (22 mm), *Aspergillus fumigatus* (15 mm), *Candida albicans* (33 mm), *Cladosporium herbarum* (25 mm), and *Fusarium oxysporum* (20 mm)	[18]
*Ruta graveolens* L. Aerial parts	*Sclerotinia sclerotiorum* (27 mm)	[90]
*Ruta graveolens* L. Aerial parts	Reduced colony forming unit (CFU) of *Bipolaris oryzae and Gerlachia oryzae*	[91]
*Ruta graveolens* L. Aerial parts	Reduced colony forming unit (CFU) of *Bipolaris oryzae and Gerlachia oryzae*	[92]
*Ruta graveolens* L. Leaves	*Monilinia fructicola* (240 μg/mL)	[54]
*Ruta graveolens* L. Leaves	*Candida albicans* (15 mm) and *Candida krusei* (17 nm)	[78]
*Ruta graveolens* L. Leaves and flowers	*Candida albicans* (MIC 1.1–2.1 μg/mL), *Candida albicans* clinical strain (MIC 1.5–2.3 μg/mL), *Candida glabrata* (MIC 1.5–2.5 μg/mL), and *Candida krusei* (MIC 1.6–2.5 μg/mL)	[55]
*Ruta montana* L. Aerial parts	*Candida albicans* (13–18 mm) and *Saccharomyces cerevisiae* (12–15 mm)	[67]
*Ruta montana* L. Aerial parts	*Candida albicans* (>40 mm)	[70]
*Ruta montana* L. Aerial parts	*Candida albicans* (22 mm, MIC > 25,000 μg/mL)	[72]
*Ruta montana* L. Aerial parts	*Aspergillus niger* (12 mm), *Candida albicans* (32 mm), *Candida dubliniensis* (24 mm), *Candida glabrata* (17 mm), *Candida* sp. (13 mm), *Candida tropicalis* (14 mm), *Cryptococcus neoformans* (20 mm), *Fusarium* sp. (14 mm), *Penicillium* sp. (15 mm), *Rhodotorula rubra* (11 mm), *Trichophyton mentagrophytes* (15 mm), and *Trichosporon* sp. (17 mm)	[71]
*Ruta montana* L. Aerial parts	*Aspergillus oryzae*, *Botrytis cinerea*, *Fusarium oxysporum*, *Fusarium solani*, and *Verticillium dahlia* with MICs 100–1100 μg/mL	[73]
*Ruta tuberculata* Forssk. Aerial parts	*Alternaria alternaria* (20 mm), *Aspergillus flavus* (17 mm), *Aspergillus fumigatus* (17 mm), *Candida albicans* (17 mm), *Cladosporium herbarum* (34 mm), and *Fusarium oxysporum* (16 mm)	[18]
*Ruta* sp. Aerial parts	*Alternaria alternata* (31 mm), *Aspergillus fumigatus* (29 mm), *Aspergillus niger* (24 mm), *Mucor mucedo* (21 mm), and *Rhizopus arrhizus* (28 mm)	[84]

## Data Availability

All relevant data have been presented as an integral part of this manuscript.
